# The transcriptional activator of the *bfp* operon in EPEC (PerA) interacts with the RNA polymerase alpha subunit

**DOI:** 10.1038/s41598-021-87586-0

**Published:** 2021-04-20

**Authors:** Cristina Lara-Ochoa, Fabiola González-Lara, Luis E. Romero-González, Juan B. Jaramillo-Rodríguez, Sergio I. Vázquez-Arellano, Abraham Medrano-López, Lilia Cedillo-Ramírez, Ygnacio Martínez-Laguna, Jorge A. Girón, Ernesto Pérez-Rueda, José Luis Puente, J. Antonio Ibarra

**Affiliations:** 1 Centro de Detección Biomolecular, Benemérita Universidad Autónoma de Puebla, Puebla, Mexico; 2 Departamento de Microbiología Molecular, Instituto de Biotecnología, Universidad Nacional Autónoma de México, Cuernavaca, Morelos Mexico; 3 Departamento de Ingeniería Celular y Biocatálisis, Instituto de Biotecnología, Universidad Nacional Autónoma de México, Cuernavaca, Morelos Mexico; 4 Laboratorio de Genética Microbiana, Departamento de Microbiología, Escuela Nacional de Ciencias Biológicas, Instituto Politécnico Nacional, Mexico City, Mexico; 5 Vicerrectoría de Investigación y Estudios de Posgrado, Benemérita Universidad Autónoma de Puebla, Puebla, Mexico; 6 Instituto de Investigaciones en Matemáticas Aplicadas y en Sistemas, Universidad Nacional Autónoma de México, Unidad Académica Yucatán, Mérida, Mexico

**Keywords:** Microbiology, Microbial genetics, Bacterial genetics

## Abstract

Enteropathogenic *E. coli* virulence genes are under the control of various regulators, one of which is PerA, an AraC/XylS-like regulator. PerA directly promotes its own expression and that of the *bfp* operon encoding the genes involved in the biogenesis of the bundle-forming pilus (BFP); it also activates PerC expression, which in turn stimulates locus of enterocyte effacement (LEE) activation through the LEE-encoded regulator Ler. Monomeric PerA directly binds to the *per* and *bfp* regulatory regions; however, it is not known whether interactions between PerA and the RNA polymerase (RNAP) are needed to activate gene transcription as has been observed for other AraC-like regulators. Results showed that PerA interacts with the alpha subunit of the RNAP polymerase and that it is necessary for the genetic and phenotypic expression of *bfpA*. Furthermore, an in silico analysis shows that PerA might be interacting with specific alpha subunit amino acids residues highlighting the direction of future experiments.

## Introduction

Enteropathogenic *Escherichia coli* (EPEC) is an *E. coli* pathovar that causes diarrhea in in children under 5 years of age and elderly people^1,2^. Typical EPEC shows two main virulence-associated phenotypes: 1) forms bacterial aggregates that attach as microcolonies to the host cell surface, a phenotype referred as “localized adherence” (LA) and 2) intimately attaches to the epithelial cells of the small intestine, generating the so-called attaching and effacing (A/E) lesion. Some EPEC strains display only the A/E phenotype and a myriad of adherence phenotypes^3,4^. Both attributes, LA and A/E, depend on two independent protein appendages that are expressed in vivo and in vitro in optimal conditions^5,6^. The LA phenotype is determined by a type IV fimbriae known as the bundle-forming pilus (BFP) encoded in the high-molecular-weight plasmid (pEAF)^5,7^. Formation of the A/E lesion is mediated by the TTSS encoded in the locus of enterocyte effacement (LEE) pathogenicity island^1,6^. BFP biosynthesis depends on the products of 14 genes likely arrayed as an operon, which transcription starts from a promoter upstream of the first gene, *bfpA*^8–10^. Transcriptional activation of *bfp* relays on PerA (also known as BfpT), a member of the AraC/XylS family of transcriptional activators encoded by the *per* (*perABC*) operon in the EAF plasmid, which also regulates its own expression^11–13^. The *per* operon also encodes PerB, which function has not yet been identified, and PerC, which has been shown to activate LEE gene expression through Ler^14–16^ and most recently to regulate EPEC metabolism for nitrate reduction under anaerobic conditions^17^. Thus, as an activator of the genes required for both virulence-related phenotypes, LA and A/E, PerA represents a central regulator of the infectious process caused by typical EPEC strains.

Most of the members of the AraC/XylS family of transcriptional activators have two domains with the following array: the amino terminus, which for some members has a signal-binding and/or dimerization role(s), and the carboxy terminus, which contains the DNA-binding domain where two helix-turn-helix (HTH) motifs are responsible for the DNA binding specificity^18–20^. PerA binds to a conserved sequence located upstream the promoters of the *perA* and *bfpA* operons^13^. Critical amino acid residues for PerA function as an activator have been identified at both domains, suggesting that in addition to those required for DNA binding, others seem to bear an important role not involving DNA binding^21^. One possibility is that these residues are involved in interactions with the RNA polymerase (RNAP). Such interactions have been documented in other members of the AraC/XylS family of transcriptional activators^22–24^. In this report we aimed to detect interactions between PerA and the RNAP. Results showed that these interactions occur with the alpha (α) subunit and confirm our previous hypothesis that PerA is a positive regulator that makes specific contacts with the transcriptional machinery and that these interactions are important for the expression of genes under PerA regulation.

## Results

### PerA interacts with the RNAP

In order to detect interactions between PerA and the RNAP we first decided to examine whether these contacts occur by using a purified version of PerA. Previously the MBP-PerA fusion allowed us to define this regulator binding sites in both *perA* and *bfpA*^13^. Thus, here we used the MBP-PerA fusion to identify interacting proteins in two related experiments: pull downs and co-purifications with cellular extracts of an EPEC strain grown in BFP-A/E inducing conditions. Before performing these experiments, the MBP-PerA purified protein preparation functionality was corroborated by electrophoretic mobility shift assays with the *perA* regulatory region. Additionally, to corroborate the conditions used to induce the expression of BFP, E2348 WT and Δ*perA* mutant strains were grown in inducing and repressing conditions (*i.e.* by adding ammonium sulfate) and the expression of BfpA was detected by Western blot (Suppl. Fig. 1A). As expected, BfpA was detected only in the WT strain inducing conditions but not under repressing conditions (*i.e.* DMEM + ammonium salt), nor in the *perA* mutant strain. MBP-PerA restore BFP expression in a Δ*perA* mutant only when no inducer was added (Suppl. Fig. 1B). This latter correlates with our previous observations that when over-produced the MBP-PerA fusion overwhelms the regulatory system and that escape transcription from the *lac* promoter is enough to produce a small amount of MBP-PerA that ultimately produce the BFP and the LA on tissue cultured cells^13^. In this regard, this small amount of MBP-PerA is not detected by Western blot with the anti-MBP antibodies (Suppl. Fig. 1B). These results showed that the conditions tested induced the expression of BfpA that depends on PerA as previously described^9,12,13^ and that the preparation of purified MBP-PerA was suitable to be used in the following experiments.

**Figure 1:**
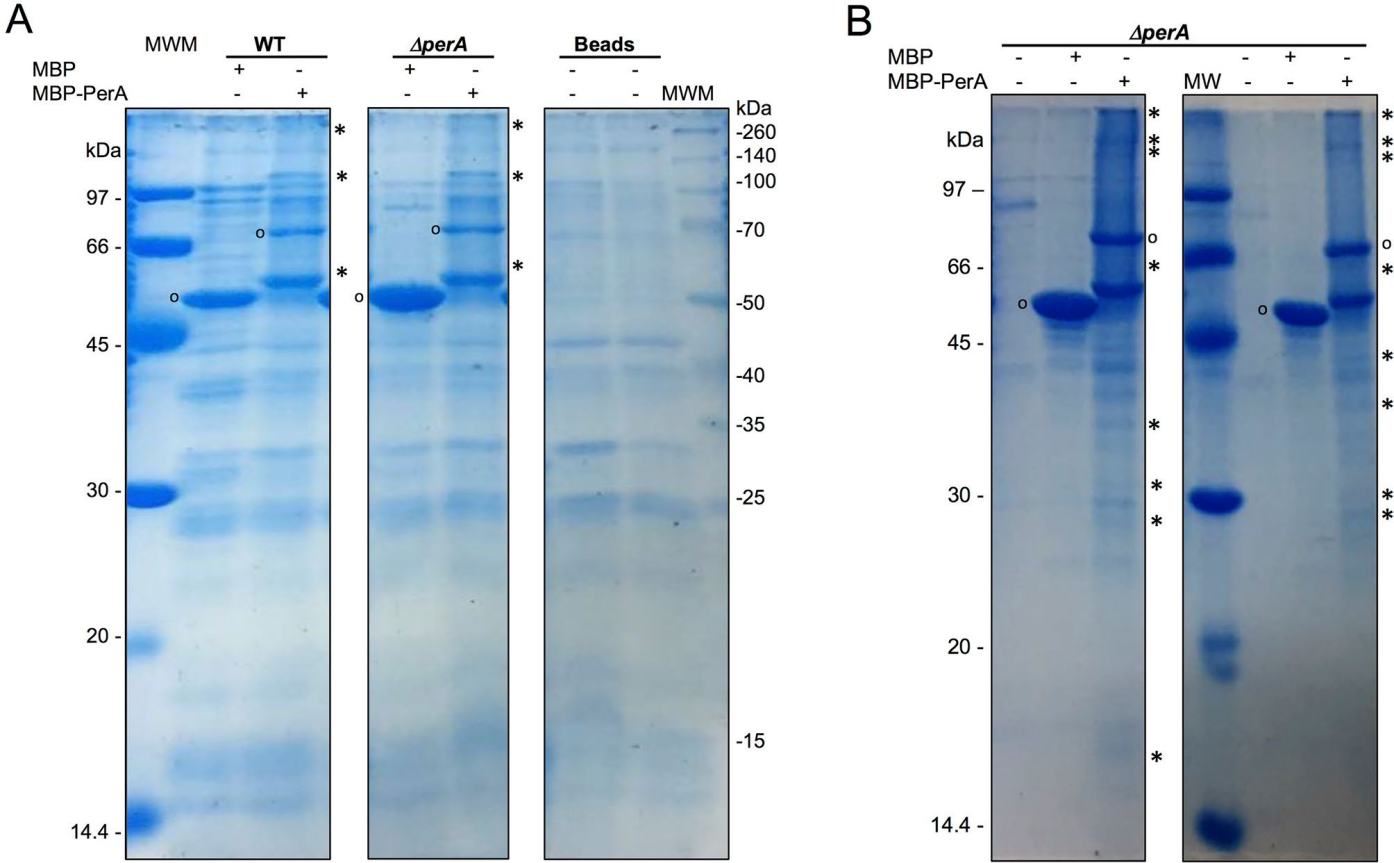
Co-purification and pull-down experiments with MBP-PerA and EPEC strain E2348/69 lysates. Protein–protein interaction experiments for pull-down (**A**) and co-purification (**B**) using either purified or over expressed MBP-PerA or MBP were performed as described in the Methods section. Samples were subjected to electrophoresis in a SDS-PAGE and then stained with Coomassie blue. Asterisks indicate proteins that were distinguished as differential when compared with the MBP control and cut for LC–MS/MS analysis and empty circles show the bands corresponding to either MBP or MBP-PerA. Shown are sections of the gels for both pull-down (**A**) and co-purification (**B**). Controls for beads are also shown. Experiments were done in triplicate and bands that showed reproducibility were submitted for analysis.

Once the experimental system was tested the MBP-PerA fusion was used for pull-down experiments with the cytoplasmic content of Δ*perA* and WT EPEC strains to detect interacting proteins with this regulator by proteomic analyses. Thus, a pull-down experiment was performed with amylose resin and interacting proteins were resolved by SDS-PAGE (Fig. 1A). Differential protein bands identified between MBP (negative control) and MBP-PerA (bait) pulled down proteins were selected on the basis of being present in the bait but not in the negative control in the three experimental replicates done and their molecular weight; this is, those bands near the expected size for the RNAP subunits were chosen. These bands were excised and analyzed by LC–MS/MS. In parallel, a co-purification approach was also used and bands were selected using a similar rationale as described above (Fig. 1B). In the co-purification procedure the plasmids encoding either MBP or MBP-PerA (Table 1) were transformed in the Δ*perA* EPEC strain and cellular extracts were obtained in inducing conditions. Co-purifed proteins were resolved in an SDS-PAGE and differential bands were identified, selected as described above and submitted for analysis by LC–MS/MS. Among the identified proteins in both experiments the β, β’, α and σ subunits of the RNAP were identified same as other proteins that will require a further analysis (Table 2 and Suppl. file 1). Taken together, these results suggest that PerA is interacting with the transcriptional machinery.

**Table 1:** Strains and plasmids used in this study.

Strain or plasmid	Genotype or description	Source or references
***E. coli strain***		
E2348/69	Wild-type EPEC O127:H6, Sm^R^	^53^
JPEP20	E2348/69 derivate, Δ*perA::kan*	^16^
MC4100	F^-^*araD139 Δ*(*argF*-*lac*)*U169 rpsL150 relA1 flb5301 deoC1 ptsF25 rbsR*	^54^
BL21(DE3) pLysS	F^-^*ompT* *gal* *dcm* *lon* *hsdS*_*B*_(*r*_*B*_^–^*m*_*B*_^–^) λ(DE3 [*lacI* *lacUV5*-*T7p07* *ind1sam7* *nin5*]) [*malB*^+^]_K-12_(λ^S^) pLysS[*T7p20* *ori*_p15A_](Cm^R^)	Invitrogen
**Plasmids**		
pMalC2xa	Vector for constructing MBP fusions	New England Biolabs
pMALT2	pMalC2xa derivate expressing MBP-PerA	^21^
pET-RpoA	pET28a derivate expressing His_6_-RpoA	^42^
pINIIIA1	pBR322 derivate cloning vector for mutants of *rpoA*	^55^
pLAX185	pINIIIA1derivate expressing wild type RpoA	^27^
pLAD235	pINIIIA1derivate expressing RpoA truncated from the carboxy end, which retain 235 amino acid	^27^
pLAD256	pINIIIA1derivate expressing RpoA truncated from the carboxy end, which retain 256 amino acid	^27^

**Table 2:** Summary of identified proteins interacting with MBP-PerA.

Estimated molecular weight (kDa)^a^	Experiment^b^	Protein identity^c^	Molecular weight (kDa)	Accession number (GenBank)	Protein coverage (%)^d^
290	Co-purification	DNA-directed RNA polymerase subunit beta'	155	WP_000653936.1	75
		DNA-directed RNA polymerase subunit beta	151	WP_000263098.1	56
140	Pull-down	DNA-directed RNA polymerase subunit beta'	155	WP_000653936.1	75
		DNA-directed RNA polymerase subunit beta	151	WP_000263098.1	71
		DNA-directed RNA polymerase sigma factor RpoD	70	WP_000437371.1	44
		ATP-dependent Clp protease ATP-binding subunit ClpA	84	WP_000934041.1	36
110	Pull-down	DNA-directed RNA polymerase subunit beta'	155	WP_000653936.1	41
		DNA-directed RNA polymerase sigma factor RpoD	70	WP_000437371.1	26
		DNA-directed RNA polymerase subunit beta	151	WP_000263098.1	20
32	Pull-down	DNA-binding transcriptional regulator KdgR	30	WP_001262188.1	58
		DeoR/GlpR family transcriptional regulator	28	WP_001296480.1	54
		DNA-directed RNA polymerase subunit alpha	37	WP_001162094.1	20
30	Pull-down	DeoR/GlpR family transcriptional regulator	28	WP_001296480.1	84
		Transcriptional regulator FNR	28	WP_000611911.1	28
		DNA-binding protein H-NS	16	WP_001287378.1	25
		DNA-directed RNA polymerase subunit alpha	37	WP_001162094.1	21

Complete dataset is in Supplementary file 1.

^a^Estimated by mobility in the SDS-PAGE and compared with the molecular weight marker.

^b^Indicates from which experiments the analyzed band comes from.

^c^Protein identification was carried out by the Proteomics facility using the *E. coli* protein database (see the Methods section for details).

^d^The number indicates percentage of coverage of the corresponding protein with the identified peptides.

### PerA interacts with the α-subunit of the RNAP

**Figure 2:**
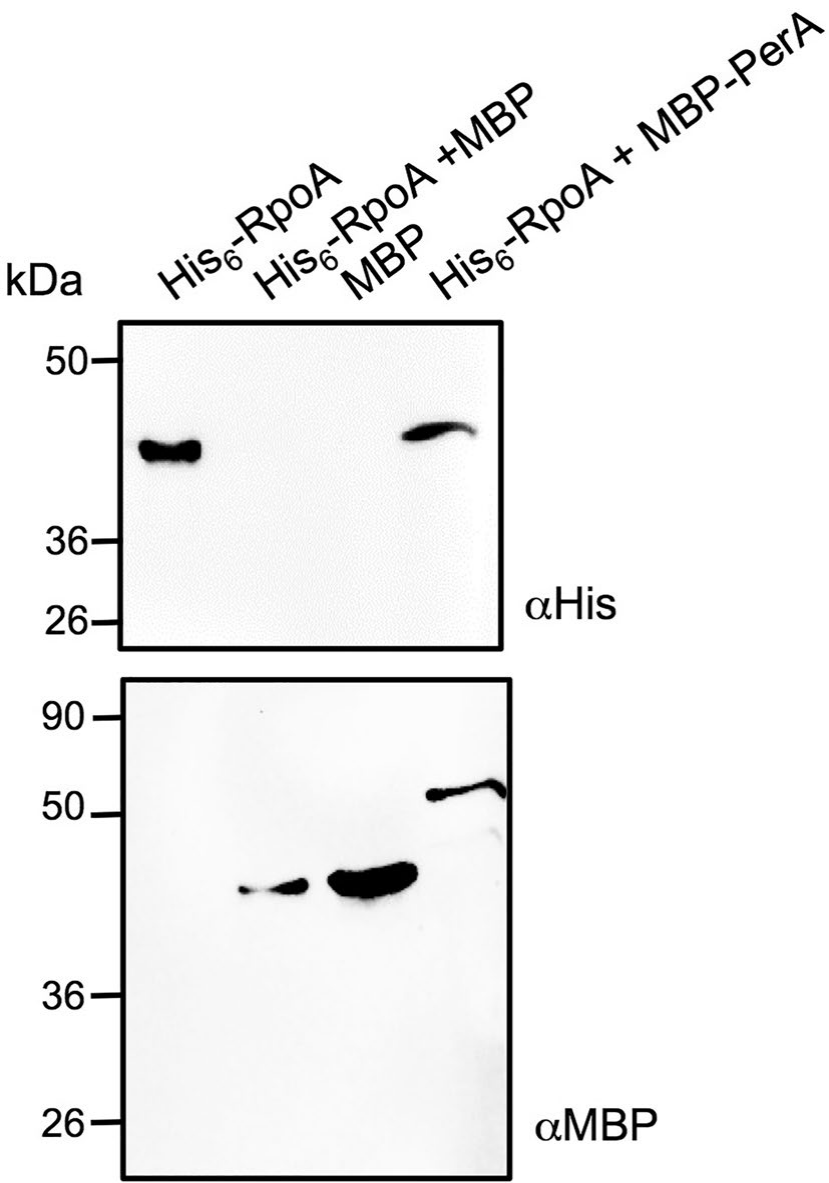
Purified MBP-PerA and His_6_-RpoA interact in vitro. A pull-down assay was performed with purified MBP or MBP-PerA proteins and purified His_6_-RpoA. The mixtures for protein–protein interactions were performed by mixing 50 µg of purified MBP or MBP-PerA and His_6_-RpoA in interaction buffer. To immobilize the bait protein the mixtures were incubated with 50 µL of amylose resin. Samples were analyzed by Western blot using an anti-MBP antibody or an anti-His_6_-HRP probe. In the first lane purified His_6-_RpoA was used as a control and in the rest of the lanes the interacting mixture is indicated. This figure is a representative of an experiment done in duplicates.

Given the results observed with the pull-down and co-purification experiments and that many bacterial transcriptional regulators in the AraC/XylS family make contacts with the alpha (α) subunit of the RNAP^18,19,22–26^, we decided to determine whether PerA interacts with the α-subunit of the RNAP (RpoA). First, a purified version of RpoA (His_6_-RpoA) was tested for interactions with MBP-PerA. As seen in Fig. 2, His_6_-RpoA was pulled down with amylose resin and identified by Western blot only when MBP-PerA was used and not with MBP. This result corroborates that both proteins interact in solution in vitro.

**Figure 3:**
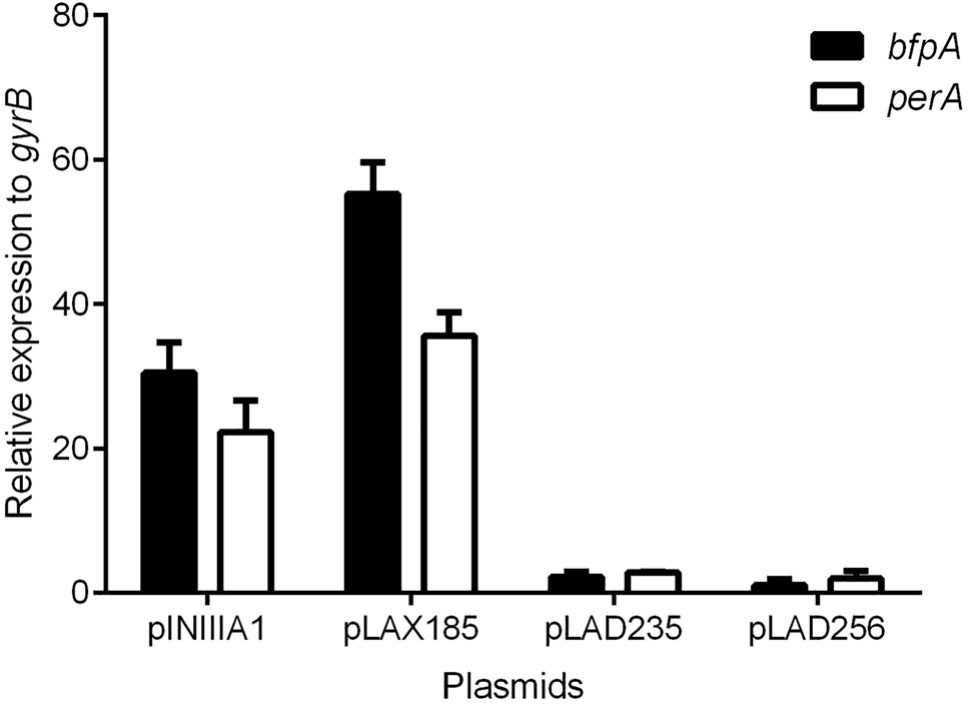
Expression of *bfpA* and *perA* is affected in RpoA CTD dominant negative mutants. Transcriptional expression of the *bfpA* and *perA* genes in EPEC WT strains transformed with pNIIIA1, pLAX185, pLAD235 and pLAD256 was detected by RT-qPCR. The strains were grown in inducing conditions in DMEM media at 37ºC supplemented with 0.25 mM IPTG to an D.O._600_ = 0.8. Graph represents the mean of two independent experiments performed in triplicates, error bars represent the standard error and the bars indicate the average of the relative expression compared to the endogenous genes *gyrB*.

In order to further confirm that PerA is interacting with RpoA in vivo two dominant negative versions of RpoA were used. These RpoA negative dominant mutants were described and kindly donated by Dr. Wilma Ross and Dr. Richard Gourse from the University of Wisconsin^27,28^. When over expressed in a bacterium these mutants compete with the wild type RpoA for the rest of the RNAP subunits during its biogenesis. When a defective RpoA is loaded in a RNAP the resulting enzyme is defective for interactions with UP elements in the DNA, but also fails to interact with transcriptional regulators that make contacts with the carboxyl domain of the α-subunit (CTD-α). Here two RpoA negative dominant mutants lacking different portions of CTD- α encoded in plasmids pLAD235 and pLAD256 (Table 1) were transformed in the wild type EPEC strain and the expression of both *perA* and *bfpA* was tested by RT-qPCR from bacterial samples taken from BFP inducing conditions (see above) (Fig. 3). The expression of both genes was observed when an empty vector (pINIIIAa, Table 1) or one encoding a wild type version of *rpoA* were tested. In contrast, the expression of *perA* and *bfpA* was abolished in both RpoA negative dominant mutants.

**Figure 4:**
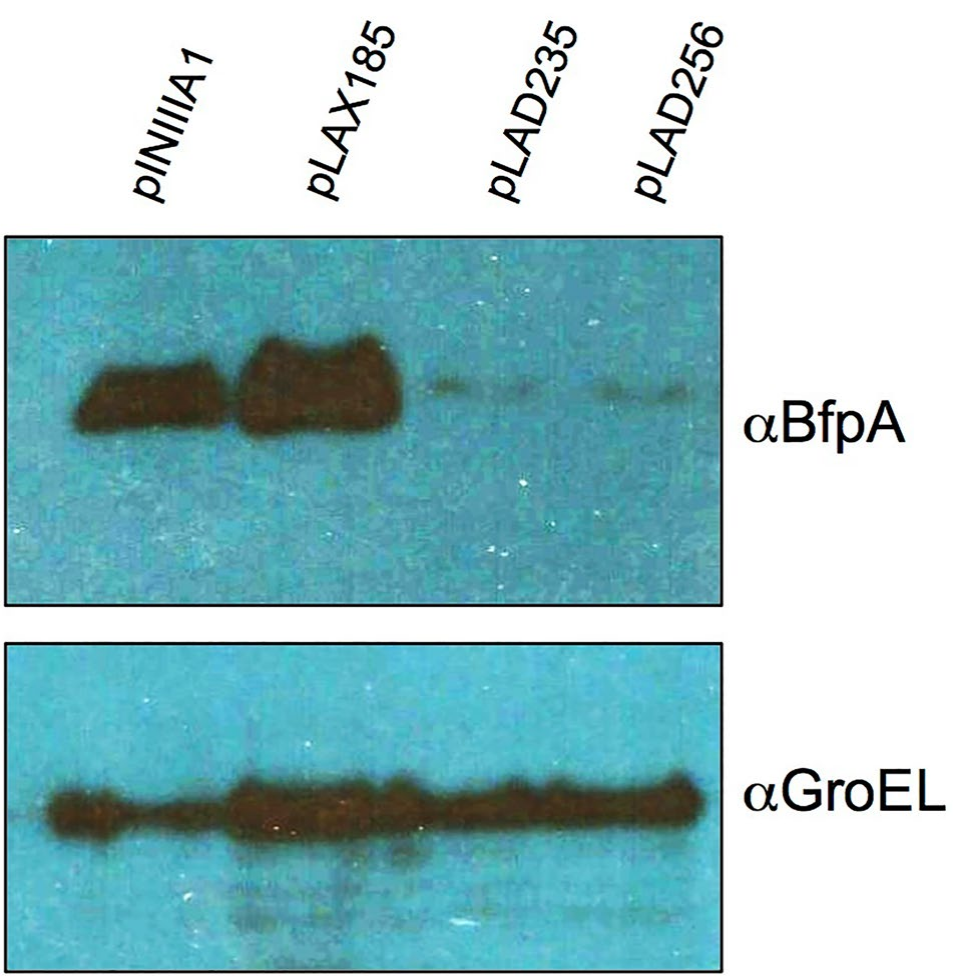
BfpA expression is defective in RpoA-CTD mutants. BfpA expression in EPEC WT strains transformed with pNIIIA1, pLAX185, pLAD235 and pLAD256 was detected by Western blot in strains grown in DMEM media at 37ºC in presence of 0.3 mM IPTG at D.O._600_ = 0.8. Detection of GroEL was done with anti-GroEL antibodies and used as loading and expression controls. This figure is a representative of an experiment done in triplicates.

In order to validate these results, the expression of BfpA was tested by Western blot in the clones expressing the RpoA negative dominant mutants. As seen in Fig. 4, BfpA was faintly detected in both RpoA mutants. Taken together these results demonstrate that the interaction of PerA and RpoA is necessary in vivo for the expression of *perA* and *bfpA*.

### PerA-RpoA interaction model

**Figure 5:**
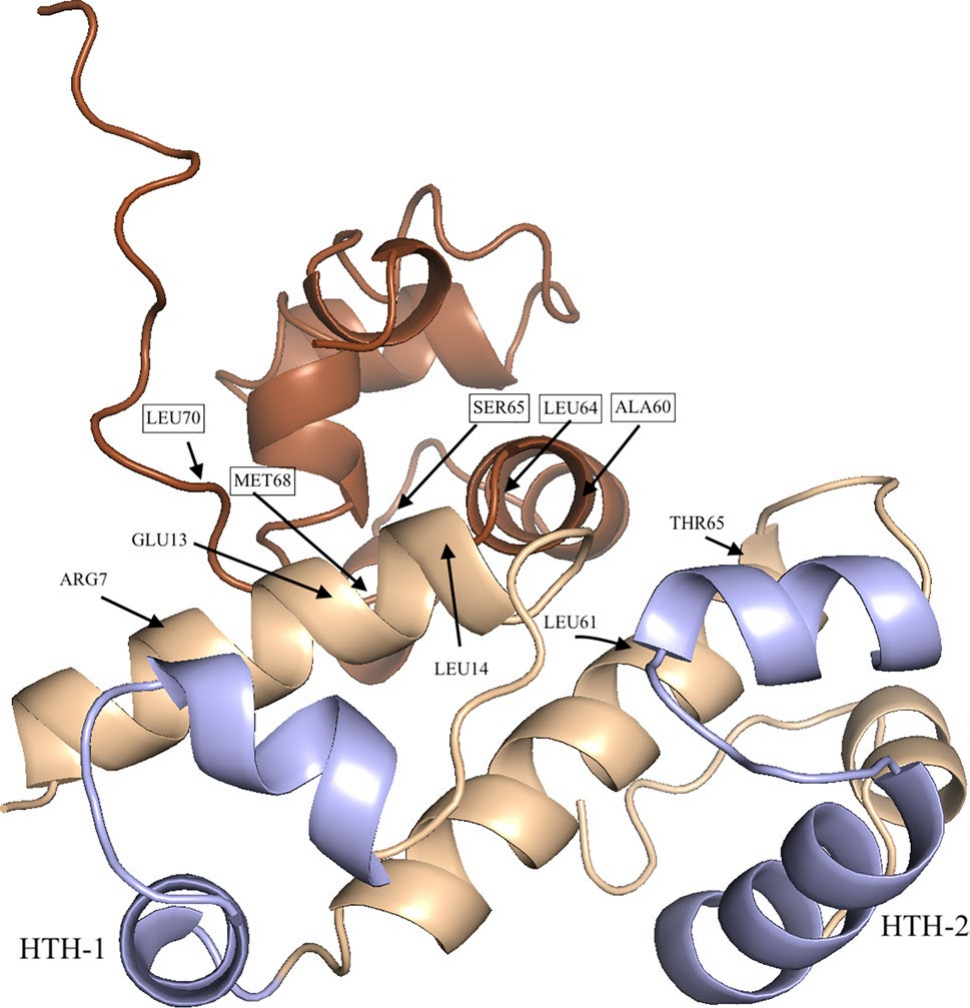
Proposed contacts between PerA and RpoA. A docking model for PerA-CTD and RpoA-CTD was generated the CPHmodels 3.2 server (http://www.cbs.dtu.dk/services/CPHmodels) using the *E. coli* transcriptional factor Rob (PDB entry 1D5Y chain A) as a template. Stereochemical quality of the model was assessed by using the Ramachandran plot at RAMPAGE program SAVES v6.0 (https://saves.mbi.ucla.edu/). The C-terminal domain of the RNAP α-subunit (PDB entry 1COO) was used to model the interaction with PerA with the HawkDock program (http://cadd.zju.edu.cn/hawkdock). PerA-CTD is shown in gold and purple and RpoA-CTD (CTD-α) in brown. Putative interacting residues are labeled as follows: RpoA residues are shown in boxes while those from PerA-CTD are not in boxes. PerA HTHs are indicated.

Given the results showing that PerA contacts CTD-α we aimed to develop a structural model of this interaction. Using a previous model generated for the PerA DNA binding domain^21^ based on the related protein Rob and the crystal structure reported for the CTD-α^29^ a contact model of the two carboxyl terminal domains was generated (Fig. 5). This docking model suggests that diverse residues could be involved in the interactions between the C-terminal domain of PerA and CTD-α. In particular, we identified a cluster of five residues (A308, L312, S313, L318, and M316) in CTD-α that could interact with PerA residues R169, E175, L176, L223, and T227. These residues exhibit a binding free energy between -1.3 and -4.9, and correspond to the top predicted and crystal models used as benchmark datasets. Indeed, these binding free energy suggest these residues have a high possibility for protein–protein interactions.

## Discussion

PerA is a central regulator for typical EPEC strains as it activates genes involved in virulence. In a previous report we have shown that this transcriptional factor is able to bind to the *bfpA* and *perA* promoter regions as a monomer^13^. Moreover, residues relevant for its function have been identified in both domains of the protein^21^. Here, our working hypothesis was that, PerA being a positive regulator that might be either a class I or a class II regulator binds to DNA and it probably makes contacts with the RNAP in a similar fashion as some members of the AraC/XylS family^30,31–36^. This idea was supported by the following experimental evidences: genes regulated by PerA are not expressed in its absence^10,11^ and that a few PerA point mutants bind to DNA but fail to fully activate transcription^21^. Here, by using two experimental approaches we showed that PerA binds to subunits of the transcriptional machinery when cellular extracts were obtained from inducing conditions. Considering that these approaches yielded several other additional interacting proteins, confirmation of PerA interactions with the RNAP was in need. The α -subunit of the RNAP is not involved in the catalytic activity of this enzyme but is able to contact UP elements and also in making contacts with transcriptional regulators, favoring transcription in both cases^26–28,37^. The dominant negative variants of RpoA together with protein–protein interactions experiments support the initial proposal that PerA and RpoA make contacts, which are needed for activation of transcription in vivo. The extent of these interactions as well as residues in each protein that play a role in this interaction will require and direct future experiments in our laboratories.

In the mean time, a 3-D docking model of RpoA and PerA carboxy-terminus domains suggests that two regions in each protein might be making contacts. In RpoA one of these regions is located in the last portion of RpoA (residues 308 to 316). This was an interesting finding as in other transcriptional regulators the so-called “265-determinant” has been shown to be relevant for these interactions. This region has been acknowledged with two roles: 1) Interacting with DNA UP elements and 2) interacting with transcriptional factors^22,26^. In the *perA* nor in the *bfpA* regulatory region consensus sequences for UP elements were found, discarding the possibility of RpoA binding to these regulatory regions. Thus, the predicted model suggests that interaction might be occurring in a different region than that previously described, which of course will need experimental assessment. Therefore PerA acts as a positive activator and not as an anti-repressor^11,15^, and it contacts the RNAP through the α-subunit. Studies performed with MarA showed that this regulator makes contacts with the α-subunit through the CTD-α, specifically with the 265-determinant, in a different manner as CRP^22^. Additionally, it seems that MarA recruits the RNAP to the promoters through a region of 14 amino acid residues in the first α-helix (α -1) containing residues that are likely surface exposed and not interacting with the DNA that might be responsible for the MarA-RNAP contacts^25^. These amino acid residues include an aromatic residue in position 14 and aliphatic residues in positions 33 and 37 for MarA. When an alignment between MarA, PerA and other well-characterized members of the family was done we observed that in these positions only V187 (corresponding to V33 in MarA) is conserved in PerA. Our prediction model suggests that PerA residues likely interacting with CTD-α are R169, E175, L176, L223, and T227; the former three are located in α 1 and the rest in α 4. In a previous study, when residues E116 and D168 were changed for alanine residues PerA was still able to bind DNA but was differentially affected in its ability to activate *perA* and *bfpA*^21^. At that time we suggested that these residues might be involved in interactions with the RNAP. PerA residue D168 is located near the region predicted by the 3-D model and residue E116 was not detected because resides outside of the C-terminus and therefore is not considered in the modeling. MelR is another member of the AraC/XylS family that has been studied for its contacts with RpoA. For this regulator it has been shown that it makes contact with multiple residues in CTD-α and that they vary depending on which promoter is being activated^23^. Moreover, MelR also makes contact with the σ-subunit^38^, which is likely to happen also in other members in this family. Lastly, CTD-α interactions have also been shown for other members of the family such as RhaR^39^, XylS and XylS1^33,40^. Thus, despite the fact that the DBD in the AraC/XylS family is widely conserved contacts with the RNAP have not been shown to many of them and we believe that these depend on how each regulator interacts with their corresponding binding site on the DNA. As for PerA, the rest of the residues suggested for the interaction will require further investigation as they represent novel potential sites of contact with CTD-α.

In summary, here we have shown that PerA is able to contact the α-subunit of the RNAP and that this interaction is necessary for the expression of *bfpA* and *perA*. These results corroborate our previous hypothesis that PerA recruits the RNAP to the promoter regions of these genes.

## Methods

### Bacterial strains and culture conditions

The strains and plasmids used in this study are listed in Table 1. Luria–Bertani (LB) broth or Dulbecco´s modified Eagle´s medium (DMEM) without sodium pyruvate, containing glucose (0.45%) and L-glutamine (584 mg/L) and supplemented with 1% LB (v/v), were used to grow cultures at 37ºC. When indicated the medium was supplemented with ammonium sulfate (20 mM). When necessary, antibiotics were added at the following concentrations: ampicillin (100 μg/ml), streptomycin (100 μg/ml), chloramphenicol (30 μg/ml) and kanamycin (30 μg/ml).

### DNA manipulations

DNA manipulations were performed using standard genetic and molecular techniques. Plasmid DNA was purified using High Pure DNA kit (Roche Scientific Inc.). The oligonucleotides used for amplification by PCR were synthesized at T4OLIGO and are listed in Supplementary Table S1. PCR reactions were performed in a 50 μl using DreamTaq Green PCR Master Mix (2X) (Thermo Fisher Scientific).

### Expression and purification of MBP-PerA and MBP

Expression and purification of the MBP-PerA and MBP proteins was done as described previously^13,21,41^. The concentration of purified proteins was determined by the Bradford method by using an albumin standard curve and analyzed in a 12% sodium dodecyl sulfate-polyacrilamide (SDS-PAGE) gel electrophoresis. Aliquots were stored at -70ºC until used.

### Expression and purification of His_6_-RpoA

The His-tagged RpoA protein was prepared from an *E. coli* strain BL21 (DE3) containing pET28-RpoA as described previously^42^ (Table 1). Protein concentration was determined by the Bradford method with the use of an albumin standard curve. Aliquots were stored at -70ºC until used.

### RT-qPCR assays

Relative expression of *bfpA* and *perA* in E2348 strains was determined by RT-qPCR as described previously^41,43^. Briefly, RNA was obtained from BFP and PerA inducing conditions described above. DNA was removed with a DNAse I RNAse-free kit (Thermo Fisher Scientific) and cDNA was obtained with a Revert Aid First Strand cDNA Synthesis kit (Thermo Fisher Scientific). qPCR was performed in a Light Cycler 480 Thermocycler (Roche). Relative expression of *bfpA* and *perA* was calculated with the ΔΔCt method using the expression of the gene coding for the B subunit of the gyrase (*gyrB*) as a normalizer. Oligos for each gene are listed in Table S1 (bfpA-RTF/bfpA-RTR, perA-RTF/perA-RTR and gyrB-RTF/gyrB-RTR, respectively). Experiments were done in triplicates and the results are the average of two independent experiments.

### Analysis of co-purified proteins

In order to find proteins interacting with PerA the MBP-PerA chimera was used to detect these interactions with a E2348/69 cell lysate by co-purification^44^. Briefly, the E2349/69 Δ*perA* strain containing either pMALT2 or pMALC2xa (Table 1) was grown to exponential phase in DMEM media as described above supplemented with ampicillin (100 μg/ml) and IPTG (0.3 mM). Cultures were pelleted by centrifugation, cells were resuspended in fresh DMEM and subjected to sonication for 10 min, combining 10-s pulses with 10-s resting cycles in a sonicator. The soluble lysate (400 µl) was incubated with amylose magnetics beads (70 µl) (New England Biolabs) and let to interact for 6 h at 4ºC. The beads with bound proteins were washed ten times with a washing buffer (137 mM NaCl, 2.7 mM KCl, 10 mM Na_2_HPO_4_ and 1.8 mM KH_2_PO_4_, pH 7.4). The elution of the bounded proteins from the beads was carried out by suspending the pelleted beads in 4X SDS Laemmli sample buffer (10 mM Tris–HCl [pH 7.4], 200 mM NaCl, 1 mM EDTA, 10 mM β-mercaptoethanol), and heating at 95ºC for 10 min. Experiments were done in triplicate and bands that showed reproducibility were submitted for analysis as follows: proteins were analyzed by SDS-PAGE and stained with Coomassie blue and protein bands of interest were excised with a new sterile blade for characterization by tryptic digest, liquid chromatography and mass spectrometry analysis (LC–MS/MS) at the Proteomics Discovery Platform of the Institut de Recherches Cliniques de Montréal (Quebec, Canada). Analysis was as done as described before with Mascot (Matrix Science, London, UK; version 2.6.0), and Scaffold (version Scaffold_4.10.0, Proteome Software Inc., Portland, OR). Peptide identifications were accepted if they could be established at greater than 95.0% probability by the Peptide Prophet algorithm^45^ with Scaffold delta-mass correction. Protein identifications were accepted if they could be established at greater than 95.0% probability and contained at least 2 identified peptides. Protein probabilities were assigned by the Protein Prophet algorithm^46^. Proteins that contained similar peptides and could not be differentiated based on MS/MS analysis alone were grouped to satisfy the principles of parsimony. Proteins sharing significant peptide evidence were grouped into clusters.

### Analysis of pulled down proteins

In order to corroborate interactions of PerA with cytoplasmic proteins from an E2348/69 cell lysate the MBP-PerA was used for pull-down experiments^47^. Purified MBP-PerA or MBP (40 µg) were immobilized with 70 µl of amylose magnetics beads (New Englands Biolabs) let to interact for 2 h at 4ºC and mixed with 400 µl of Δ*perA* and WT E2348/69 soluble cell extracts for 4 h in agitation at 4ºC. The beads with bounded proteins were treated as described above. The protein bands of interest were excised for characterization by LC/MS/MS at the Proteomics Discovery Platform of the Institut de Recherches Cliniques de Montréal (Quebec, Canada). Samples were selected from three replicates and analyzed as described in the previous section.

### MBP-PerA/His_6_-RpoA interactions (pull down)

Pull-down experiments were performed with purified MBP, MBP-PerA and His_6_-RpoA proteins. Two reaction mixtures were done: A negative control with MBP and His_6_-RpoA and tests with MBP-PerA and His_6_-RpoA; both were done by using 50 µg of each protein in a 2X interaction buffer (100 mM NaH_2_PO_4_, 600 mM NaCl, 40 mM imidazol, 0.5% NP-40 and 20% glycerol, pH 8.0)^48^. Proteins were let to interact for 30 min on ice, then 50 µl of amylose resin or Ni–NTA agarose beads were added to each mixture and let to interact for 2 h in agitation at ~ 4 °C. Beads were centrifuged at 2,000 × *g* for 2 min, amylose beads were washed three times with cold washing buffer and Ni–NTA beads were washed with a low concentration imidazole buffer (40 mM). After the last washing step supernatant was removed carefully and then 20 µl of 4X SDS Laemmli sample buffer were added. Samples were resolved in a 12% SDS-PAGE and stained with Coomassie blue. Western blot was performed by transferring the gel to a PVDF membrane and by following a previously described protocol with either anti-MBP antibodies (New England Biolabs) or an anti-His6 HRP probe (Invitrogen). Western blot was developed by using chemiluminescence kit (Invitrogen) and observed in a Chemidoc imaging system (Bio-Rad).

### Western blotting

For detection of BfpA a sample of 3 ml was taken from bacterial cultures, lysed and the cellular extract used to detect this protein by Western blot. Cells were resuspended in 300 µl of a urea solution (8 M) and subjected to sonication. These extracts were combined in Laemmli buffer, boiled, subjected to SDS-PAGE (12% polyacrylamide), and transferred to 0.22-μm-pore-size PVDF membranes. Membranes were blocked with 10% nonfat milk and incubated with anti-BfpA (1:10,000), anti-MBP (1:2,000) or anti-GroEL (1:80,000) antibodies. Membranes were then washed with PBS-Tween 20 (0.05%), and a 1:10,000 dilution of horseradish peroxidase-conjugated goat anti-rabbit antibody (Pierce) was added. Membranes were developed by using a SuperSignal West Pico PLUS Chemiluminescent Substrate kit (Thermo Fisher Scientific) according to the manufacturer instructions.

### Equipment and settings

Stained SDS-PAGE gels images for Fig. 1 were acquired with a cell phone camera. Western blot images in Fig. 2 were acquired in a ChemiDoc imaging system (Bio-Rad) by using the automatic setting. Later images were converted to a PDF, JPEG or TIFF files with the ImageLab software ver. 6.1 (Bio-Rad). Western blot images in Fig. 4 and Supplementary Fig. 1 were acquired by exposure to a film (Kodak X-Omat LS) and later the selected exposures were either scanned or photographed as mentioned above. Changes in brightness and contrast when considered necessary were done either with the Preview tool in a Mac computer or with Microsoft office picture manager in a Windows computer.

### Structural modeling of PerA and the α-subunit C-terminal domains

A model of the three-dimensional structure of PerA (P43459) was built with the CPHmodels 3.2 server (http://www.cbs.dtu.dk/services/CPHmodels)^49^. The *E. coli* transcriptional factor Rob (PDB entry 1D5Y chain A) was used as a template. To refine the model, we used the 3Drefine server (http://sysbio.rnet.missouri.edu/3Drefine/index.html)^50^. Finally, for assessing the quality of the protein model, we validated its stereochemical quality of the resulting three-dimensional model by using the Ramachandran plot at RAMPAGE SAVES v6.0 program (https://saves.mbi.ucla.edu/)^51^. The Ramachandran plot, showed that 96.3% of the residues were located in favored regions and 3.7% in allowed regions. All the bond distances, angles and dihedrals fulfill the normal limits for polypeptide chains. The resulting model includes 111 of the 274 residues of PerA. The C-terminal domain of the RNAP α-subunit (PDB entry 1COO) was used to model the interaction with PerA. To this end, we used the program HawkDock (http://cadd.zju.edu.cn/hawkdock), designed to predict protein–protein interactions^52^. The binding free energy of the complex was of -20.15 (kcal/mol), and the top five residues per structure were mapped into the model. Finally, the model was displayed in the PyMOL Molecular Graphics System, Version 2.0 Schrödinger, LLC (https://pymol.org/).

### Statistical analysis

Statistical analysis was performed in Excel or GraphPad Prism version 5 by using a paired-sample Student’s *t*-test. A significant difference was considered when *P* < 0.05.

## Supplementary Information

Supplementary Information
