# Supplementary material for: The transcriptional activator of the bfp operon in EPEC (PerA) interacts with the RNA polymerase alpha subunit
Source: Sci Rep. 2021 Apr 20;11:8541. doi: 10.1038/s41598-021-87586-0 (PMC8058060; doi:10.1038/s41598-021-87586-0)
Supplement: Supplementary file 1 — Supplementary Information [file 41598_2021_87586_MOESM1_ESM.pdf]

Supplementary material for the manuscript:

The transcriptional activator of the *bfp* operon in EPEC (PerA) interacts with the RNA polymerase alpha subunit

Cristina Lara-Ochoa, Fabiola González-Lara, Luis E. Romero-González, Juan B. Jaramillo-Rodríguez, Sergio I. Vázquez-Arellano, Abraham Medrano-López, Lilia Cedillo-Ramírez, Ygnacio Martínez-Laguna, Jorge A. Girón, Ernesto Pérez-Rueda, José Luis Puente, and J. Antonio Ibarra

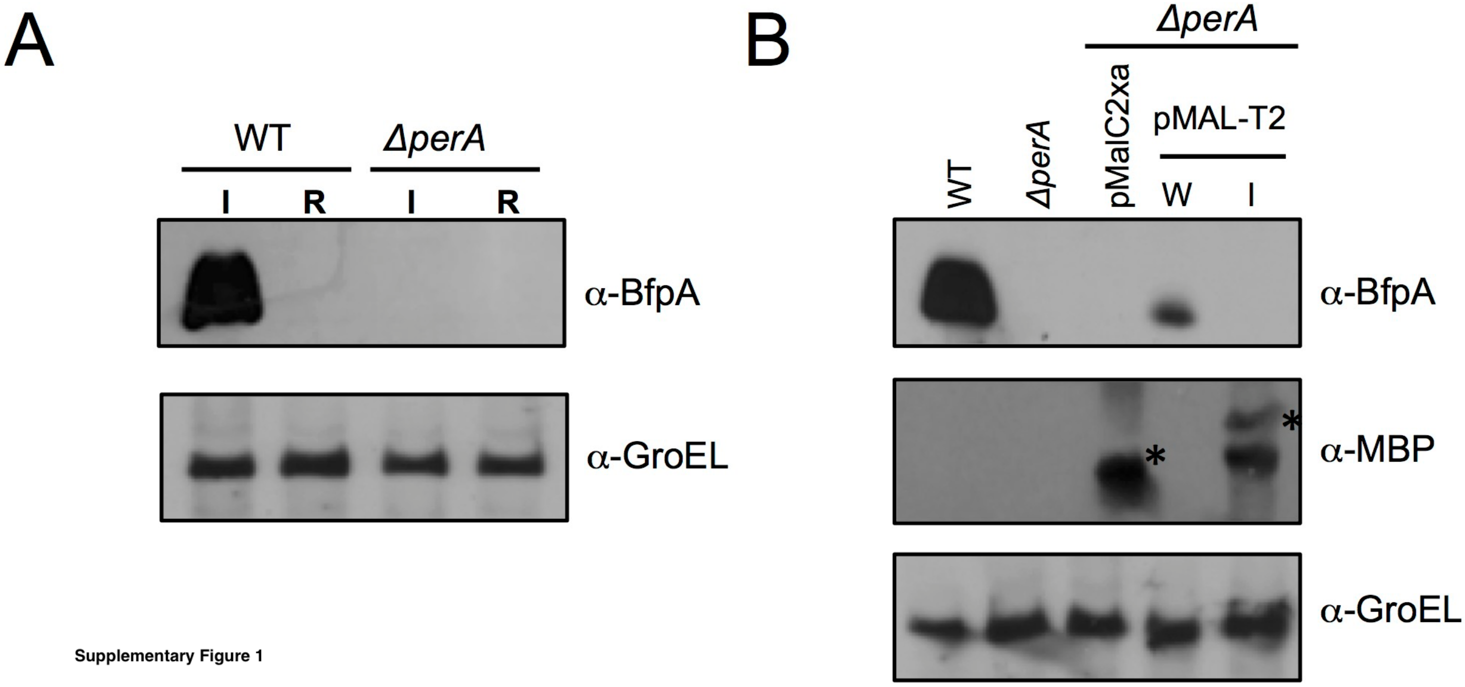

Supplementary Figure 1. Evaluation of expression conditions of BfpA for pull-down and co-purification experiments. A) E2348 WT and perA mutant strains were grown in inducing (I) and repressing conditions (R) (by adding ammonium sulfate). B) E2348 perA mutant strain with plasmids pMALC2xa or pMALT2 (Table 1) were grown in inducing conditions with (I) or without (W) IPTG. Shown are Western blottings with anti-BfpA or anti-MBP antibodies. Detection of GroEL was done with anti-GroEL antibodies and used as loading control. Asterisks indicate proteins MBP or MBP-PerA.

Table S1. Primers used in this study.

Primer	Sequence (5’-3’)	Target gene	Use
perA-RTF	cactcattgggacatgg	*perA*	RT-qPCR
perA-RTR	ttcattgaggttcgcag		
bfpA-RTF	gggcagaccagttatag	*bfpA*	RT-qPCR
bfp-RTR	agtaaccaaatgcggtg		
gyrB-RTF	accattcacgccgataactc	*gyrB*	RT-qPCR
gyrB-RTR	gggcgtttactaccgaaaca		

Supplementary File 1. Results of proteins analysis. Contains an Excel file with results from the analysis with the Scaffold program of the LC-MS/MS from proteins obtained in Figure 1. Shows identity of bands by comparing the results to the E. coli database and also the peptides counts for each identified protein.

Supplementary file 1

PerA, the transcriptional activator of the bfp operon in tEPEC, interacts with the RNA polymerase alpha subunit

Cristina Lara-Ochoa, Fabiola González-Lara, Luis E. Romero-González, Juan B. Jaramillo-Rodríguez, Sergio I. Vázquez-Arellano, Abraham Medrano-López, José Luis Puente, Lilia Cedillo-Ramírez, Ygnacio Martínez-Laguna, Jorge A. Girón, Ernesto Pérez-Rueda and J. Antonio Ibarra

Results of proteins analysis.

Contains an Excel file with results from the analysis with the Scaffold program of the LC-MS/MS from proteins obtained in Figure 1. Shows identity of bands by comparing the results to the E. coli database and also the peptides counts for each identified protein.

Experiment: Cristina_20170428_Refseq_E_Coli_str_E2348-69_20170601_All_Bands_SCAFF4
Database Set: 1 Database
Database Name: the Refseq_E_Coli_str_E2348-69_20170602 database
Version: unknown
Taxonomy: All Entries
Number of Proteins: 5422
Does database contain common contaminants?: unknown
Search Engine Set: 1 Search Engine
Search Engine: Mascot
Version: 2.6.0
Samples: All Samples
Fragment Tolerance: 0.60 Da (Monoisotopic)
Parent Tolerance: 10.0 PPM (Monoisotopic)
Fixed Modifications: +57 on C (Carbamidomethyl)
Variable Modifications: +16 on M (Oxidation)
Database: the Refseq_E_Coli_str_E2348-69_20170602 database (unknown version, 5422 entries)
Digestion Enzyme: Trypsin
Max Missed Cleavages: 2
Probability Model: Analysis performed prior to Scaffold 4
Scaffold: Version: Scaffold_4.10.0
Modification Metadata Set: 1541 modifications
Source: C:\Program Files\Scaffold 4\parameters\unimod.xml
Comment:
Protein Grouping Strategy: Experiment-wide grouping with protein cluster analysis
Peptide Thresholds: 95.0% minimum
Protein Thresholds: 95.0% minimum and 2 peptides minimum
Peptide FDR: 0.0% (Decoy)
Protein FDR: 0.0% (Decoy)
GO Annotation Source(s):
Pathway Annotation Source(s): Unknown
Alternate ID Source(s):

							Experiment (P: pull down; C: co-purification)		C	C	C	C		P		P	C	P		P
All_Bands							Apparent band size (kDa)		13	23	38	290		30		32	52	140		110
#	Visible?	Starred?	Identified Proteins (439)	Accession Number	MW	Protein	Taxonomy	00-BLK befor	01-Band 9	02-Band 10	03-Band 7	04-Band 6	05-BLK befor	06-Band 5	07-BLK befor	08-Band 3	09-Band 4	10-Band 1	11-BLK befor	12-Band 2
1	TRUE	Empty	MULTISPECIES: maltose ABC transporte	WP_000695389.1	43 kDa		unknown	0	60	250	120	378	30	75	12	82	273	161	28	363
2	TRUE	Empty	porin OmpA [Escherichia coli]	WP_012578862.1	37 kDa		unknown	0	0	190	32	0	3	84	6	636	148	44	90	1
3	TRUE	Empty	intimin [Escherichia coli]	WP_000627890.1	102 kDa		unknown	0	0	0	0	1	0	4	0	20	86	352	14	53
4	TRUE	Empty	MULTISPECIES: heat-shock protein IbpA	WP_001243437.1	16 kDa		unknown	0	380	70	22	11	24	0	5	0	0	0	3	0
5	TRUE	Empty	MULTISPECIES: 30S ribosomal protein S	WP_000529945.1	26 kDa		unknown	0	4	48	16	22	0	70	3	47	23	43	0	38
6	TRUE	Empty	DNA-directed RNA polymerase subunit	WP_000653936.1	155 kDa		unknown	0	0	2	6	219	0	0	0	2	7	181	2	77
7	TRUE	Empty	MULTISPECIES: DNA-directed RNA poly	WP_000263098.1	151 kDa		unknown	0	1	1	0	144	0	0	0	0	0	187	3	40
8	TRUE	Empty	carbamoyl-phosphate synthase large c	WP_001126342.1	118 kDa		unknown	0	0	0	3	59	0	0	0	0	0	253	6	45
9	TRUE	Empty	MULTISPECIES: 50S ribosomal protein L	WP_000301864.1	30 kDa		unknown	0	0	13	2	11	0	53	3	74	15	14	1	15
10	TRUE	Empty	MULTISPECIES: 30S ribosomal protein S	WP_000246882.1	27 kDa		unknown	0	0	5	0	0	0	230	25	16	1	0	3	0
11	TRUE	Empty	MULTISPECIES: PTS mannose transport	WP_000228655.1	31 kDa		unknown	0	0	5	0	0	0	113	5	88	11	1	2	1
12	TRUE	Empty	MULTISPECIES: translation elongation f	WP_000031783.1 (+1	43 kDa		unknown	0	2	8	8	5	0	39	1	50	80	62	10	35
13	TRUE	Empty	MULTISPECIES: DeoR/GlpR family trans	WP_001296480.1	28 kDa		unknown	0	0	0	0	0	0	249	9	14	0	0	3	0
14	TRUE	Empty	bifunctional acetaldehyde-CoA/alcohol	WP_000301661.1	96 kDa		unknown	0	0	0	4	15	0	8	0	3	72	109	3	113
15	TRUE	Empty	hypothetical protein [Escherichia coli]	WP_001346246.1	6 kDa		unknown	0	11	21	16	79	8	8	3	11	38	28	6	49
16	TRUE	Empty	AraC family transcriptional regulator [E	WP_000481009.1	10 kDa		unknown	0	17	26	14	39	0	9	0	10	7	30	0	60
17	TRUE	Empty	MULTISPECIES: heat-shock protein IbpB	WP_001243431.1	16 kDa		unknown	0	148	21	2	1	8	0	2	0	0	0	1	0
18	TRUE	Empty	MULTISPECIES: translation initiation fa	WP_000133040.1	97 kDa		unknown	0	0	0	0	2	0	0	0	1	2	91	4	89
19	TRUE	Empty	MULTISPECIES: elongation factor G [Pr	WP_000124700.1	78 kDa		unknown	0	0	1	2	6	0	2	0	2	6	123	10	35
20	TRUE	Empty	MULTISPECIES: aldehyde dehydrogena	WP_000153502.1	36 kDa		unknown	0	0	4	67	3	0	6	0	7	64	13	0	2
21	TRUE	Empty	MULTISPECIES: 30S ribosomal protein S	WP_000135224.1	23 kDa		unknown	0	0	58	2	0	0	45	0	39	6	10	0	2
22	TRUE	Empty	phosphoribosylformylglycinamidine sy	WP_000970167.1	141 kDa		unknown	0	0	0	0	40	0	0	0	0	0	116	9	0
23	TRUE	Empty	MULTISPECIES: asparagine synthetase	WP_000337076.1	63 kDa		unknown	0	0	0	0	0	0	0	0	0	105	0	0	0
24	TRUE	Empty	MULTISPECIES: membrane protein inse	WP_000378258.1	62 kDa		unknown	0	0	0	0	0	0	0	0	0	95	0	0	0
25	TRUE	Empty	MULTISPECIES: formate acetyltransfera	WP_001292820.1	85 kDa	TRUE	unknown	0	0	2	1	2	0	1	0	1	11	99	5	12
26	TRUE	Empty	phosphoribosylformylglycinamidine cy	WP_001339839.1	37 kDa		unknown	0	0	0	94	1	0	0	0	0	5	0	0	0
27	TRUE	Empty	MULTISPECIES: LysR family transcriptio	WP_000776253.1	36 kDa		unknown	0	0	0	119	0	2	0	0	0	2	0	0	0
28	TRUE	Empty	2-oxoglutarate dehydrogenase subunit	WP_001181513.1	105 kDa		unknown	0	0	0	0	0	0	0	0	0	0	9	0	88
29	TRUE	Empty	MULTISPECIES: 30S ribosomal protein S	WP_000140327.1	61 kDa		unknown	0	0	0	0	0	0	1	0	3	18	22	0	48
30	TRUE	Empty	secretion protein EspD [Escherichia col	WP_000935757.1	39 kDa		unknown	0	0	0	0	0	0	53	0	12	12	11	0	35
31	TRUE	Empty	MULTISPECIES: hydrogenase-2 large ch	WP_000083065.1	62 kDa		unknown	0	1	0	0	0	0	0	0	0	93	1	0	0

32	TRUE	Empty	exoribonuclease R [Escherichia coli]	WP_000076339.1	92 kDa		unknown	0	0	0	0	0	0	1	0	0	0	72	2	29
33	TRUE	Empty	MULTISPECIES: 50S ribosomal protein	WP_000579833.1	22 kDa		unknown	0	1	63	1	0	0	13	0	26	1	4	0	0
34	TRUE	Empty	MULTISPECIES: cytochrome bd-I ubiqu	WP_000884361.1	58 kDa		unknown	0	0	0	0	2	0	2	0	0	16	67	0	22
35	TRUE	Empty	lac repressor [Escherichia coli]	WP_000805887.1	39 kDa		unknown	0	0	48	35	14	0	9	0	9	0	0	0	0
36	TRUE	Empty	MULTISPECIES: glutamine synthetase [	WP_001271717.1	52 kDa		unknown	0	0	0	4	1	0	0	0	0	106	1	0	0
37	TRUE	Empty	MULTISPECIES: pyruvate dehydrogena	WP_000963544.1	66 kDa		unknown	0	0	3	9	2	0	2	0	0	6	69	10	10
38	TRUE	Empty	UDP-glucose 4-epimerase GalE [Escher	WP_000234389.1	38 kDa		unknown	0	0	6	83	1	0	2	0	0	2	0	0	0
39	TRUE	Empty	trifunctional transcriptional regulator/	WP_012578870.1	144 kDa		unknown	0	0	0	0	0	0	0	0	0	0	90	0	19
40	TRUE	Empty	polyribonucleotide nucleotidyltransfer	WP_012579005.1	77 kDa		unknown	0	0	0	1	3	0	0	0	0	5	82	4	5
41	TRUE	Empty	ribonuclease E [Escherichia coli]	WP_000827393.1	118 kDa		unknown	0	0	0	0	48	0	0	0	0	5	45	0	14
42	TRUE	Empty	MULTISPECIES: molecular chaperone G	WP_000729117.1	57 kDa		unknown	0	0	0	0	4	0	1	0	0	71	13	0	10
43	TRUE	Empty	MULTISPECIES: pyruvate kinase I [Prot	WP_001295403.1	51 kDa		unknown	0	0	0	0	0	0	0	0	0	103	0	2	0
44	TRUE	Empty	methionine synthase [Escherichia coli]	WP_000095940.1	136 kDa		unknown	0	0	0	0	1	0	0	0	0	0	82	6	8
45	TRUE	Empty	MULTISPECIES: beta-hydroxyacyl-ACP	WP_000210739.1	17 kDa		unknown	0	60	6	1	1	0	10	2	0	0	4	1	0
46	TRUE	Empty	MULTISPECIES: amidophosphoribosyltr	WP_000334221.1	57 kDa		unknown	0	0	0	0	0	0	0	0	0	96	0	0	0
47	TRUE	Empty	bifunctional biotin--[acetyl-CoA-carbox	WP_000654597.1	35 kDa		unknown	39	4	1	1	0	15	0	7	0	0	0	3	0
48	TRUE	Empty	MULTISPECIES: rod shape-determining	WP_000913396.1	37 kDa		unknown	0	2	7	54	2	0	8	0	3	11	0	0	0
49	TRUE	Empty	MULTISPECIES: outer membrane prote	WP_001240896.1	91 kDa		unknown	0	0	0	0	0	0	0	0	0	1	75	1	5
50	TRUE	Empty	MULTISPECIES: ATP synthase subunit b	WP_000190506.1	50 kDa		unknown	0	0	9	8	5	0	8	0	6	30	9	0	4
51	TRUE	Empty	MULTISPECIES: lipoyl synthase [Bacteri	WP_000042632.1	36 kDa		unknown	0	0	2	56	0	0	5	0	1	1	0	0	0
52	TRUE	Empty	MULTISPECIES: DNA-binding response	WP_001194358.1	27 kDa		unknown	0	0	0	0	0	0	53	1	0	0	0	0	0
53	TRUE	Empty	MULTISPECIES: 30S ribosomal protein	WP_000829818.1	15 kDa		unknown	0	36	2	0	1	0	15	0	3	4	2	0	2
54	TRUE	Empty	MULTISPECIES: pyruvate dehydrogena	WP_000003820.1	100 kDa		unknown	0	0	2	2	15	0	3	0	0	0	23	0	31
55	TRUE	Empty	maltodextrin phosphorylase [Escherich	WP_000081904.1	91 kDa		unknown	0	0	1	0	0	0	0	0	0	0	73	0	1
56	TRUE	Empty	MULTISPECIES: oligopeptide ABC trans	WP_001297114.1	61 kDa		unknown	0	0	0	0	0	0	0	0	0	70	0	0	0
57	TRUE	Empty	MULTISPECIES: RNA polymerase sigma	WP_000437371.1	70 kDa		unknown	0	0	0	0	2	0	0	0	0	0	43	0	19
58	TRUE	Empty	MULTISPECIES: EscC/YscC/HrcC family	WP_000723931.1	56 kDa		unknown	0	0	0	0	0	0	0	0	0	79	0	0	0
59	TRUE	Empty	MULTISPECIES: RNA chaperone ProQ [	WP_000431376.1	26 kDa		unknown	0	0	0	0	0	0	41	3	0	0	0	0	0
60	TRUE	Empty	MULTISPECIES: multidrug efflux RND tr	WP_001132480.1	114 kDa		unknown	0	0	0	0	3	0	0	0	0	0	62	0	5
61	TRUE	Empty	selenide, water dikinase SelD [Escheric	WP_001339697.1	37 kDa		unknown	0	0	0	60	0	0	0	0	0	0	0	0	0
62	TRUE	Empty	glycogen-branching enzyme [Escherich	WP_001283725.1	84 kDa		unknown	0	0	0	0	0	0	3	0	0	3	50	1	0
63	TRUE	Empty	MULTISPECIES: phosphoribosylaminoir	WP_001295467.1	27 kDa		unknown	0	0	52	2	2	0	3	0	0	0	0	0	0
64	TRUE	Empty	MULTISPECIES: septum site-determinir	WP_000101055.1	30 kDa		unknown	0	2	1	0	0	0	35	1	21	0	0	0	0
65	TRUE	Empty	MULTISPECIES: 30S ribosomal protein	WP_001216676.1	15 kDa		unknown	0	57	0	0	0	0	4	0	0	0	0	0	0
66	TRUE	Empty	MULTISPECIES: dihydrolipoyl dehydrog	WP_000102485.1	51 kDa		unknown	0	0	1	3	7	0	2	0	4	34	8	0	7
67	TRUE	Empty	DNA polymerase I [Escherichia coli]	WP_000249992.1	103 kDa		unknown	0	0	0	0	0	0	0	0	0	0	11	0	56
68	TRUE	Empty	MULTISPECIES: bifunctional aspartokin	WP_001264707.1	89 kDa		unknown	0	0	2	0	5	0	0	0	0	0	49	0	0
69	TRUE	Empty	MULTISPECIES: protease modulator Hf	WP_001232412.1	38 kDa		unknown	0	0	0	47	0	0	0	0	0	0	0	0	0
70	TRUE	Empty	MULTISPECIES: 50S ribosomal protein	WP_001085926.1	15 kDa		unknown	0	49	3	0	0	0	0	0	0	0	0	0	0
71	TRUE	Empty	MULTISPECIES: trigger factor [Proteob	WP_001198386.1	48 kDa		unknown	0	0	0	0	0	0	0	0	0	63	0	0	0
72	TRUE	Empty	phosphate acetyltransferase [Escherich	WP_000086706.1	77 kDa		unknown	0	1	0	2	8	0	1	0	2	2	41	0	4
73	TRUE	Empty	MULTISPECIES: PurR family transcriptio	WP_000190985.1	38 kDa		unknown	0	0	0	49	0	0	0	0	0	1	0	0	0
74	TRUE	Empty	hypothetical protein [Escherichia coli]	WP_000032717.1	31 kDa		unknown	0	0	0	0	0	0	12	0	6	0	0	0	0
75	TRUE	Empty	MULTISPECIES: 30S ribosomal protein	WP_000246815.1	14 kDa		unknown	0	19	6	0	0	0	6	0	2	2	3	0	3
76	TRUE	Empty	MULTISPECIES: 30S ribosomal protein	WP_001029684.1	14 kDa		unknown	0	39	3	0	2	0	2	0	0	0	4	0	3
77	TRUE	Empty	sulfate adenylyltransferase [Escherichi	WP_001090352.1	53 kDa		unknown	0	0	0	0	0	0	0	0	0	57	0	0	0
78	TRUE	Empty	MULTISPECIES: DEAD/DEAH box family	WP_001295553.1	71 kDa		unknown	0	0	5	0	0	0	3	0	4	13	13	0	22
79	TRUE	Empty	MULTISPECIES: 50S ribosomal protein	WP_000613955.1	14 kDa		unknown	0	45	0	0	0	0	0	0	0	0	0	0	0
80	TRUE	Empty	MULTISPECIES: glucose-6-phosphate is	WP_000789981.1	62 kDa		unknown	0	0	0	0	0	0	0	0	0	49	0	0	0
81	TRUE	Empty	transcription-repair coupling factor [Es	WP_001340041.1	130 kDa		unknown	0	0	0	2	1	0	0	0	0	2	54	0	0
82	TRUE	Empty	tyrosine-protein kinase etk [Escherichi	WP_000208660.1	81 kDa		unknown	0	0	0	0	0	0	1	0	0	0	48	0	0
83	TRUE	Empty	DeoR family transcriptional regulator [	WP_000450116.1	29 kDa		unknown	0	0	0	0	0	0	35	1	0	0	0	0	0
84	TRUE	Empty	MULTISPECIES: 30S ribosomal protein	WP_000062611.1	14 kDa		unknown	0	39	0	0	0	0	0	0	0	0	0	0	0
85	TRUE	Empty	PTS mannose transporter subunit EIIAE	WP_000150547.1	35 kDa		unknown	0	0	0	2	0	0	3	0	6	37	0	0	0
86	TRUE	Empty	MULTISPECIES: N-acetylmuramoyl-L-al	WP_000102892.1	31 kDa		unknown	0	0	0	0	0	0	32	0	0	0	0	0	0
87	TRUE	Empty	MULTISPECIES: pyruvate dehydrogena	WP_000331776.1	29 kDa		unknown	0	0	0	0	0	0	41	1	0	0	0	0	0
88	TRUE	Empty	NADP-dependent malic enzyme [Esche	WP_000342632.1	82 kDa		unknown	0	0	0	0	16	0	0	0	0	1	28	0	1
89	TRUE	Empty	serine protease EspC [Escherichia coli]	WP_001034000.1	141 kDa		unknown	0	0	0	0	0	0	27	0	23	0	0	0	0
90	TRUE	Empty	MULTISPECIES: N-acetyl-alpha-D-gluco	WP_000999466.1	37 kDa		unknown	0	0	1	48	0	0	0	0	0	0	0	0	0
91	TRUE	Empty	MULTISPECIES: excinuclease ABC subu	WP_000357763.1	104 kDa		unknown	0	0	0	0	5	0	0	0	0	0	8	0	36
92	TRUE	Empty	phosphoporin PhoE [Escherichia coli]	WP_000977937.1	39 kDa	TRUE	unknown	0	0	7	13	0	0	16	0	8	1	1	0	0
93	TRUE	Empty	MULTISPECIES: ATP synthase subunit a	WP_001176745.1	55 kDa		unknown	0	0	3	1	3	0	1	0	0	29	6	0	2
94	TRUE	Empty	GMP synthase (glutamine-hydrolyzing)	WP_000138264.1	59 kDa		unknown	0	0	0	0	0	0	0	0	0	48	0	0	0
95	TRUE	Empty	MULTISPECIES: alpha/beta hydrolase [	WP_000704512.1	32 kDa		unknown	0	0	0	0	0	0	0	0	47	0	0	0	0
96	TRUE	Empty	MULTISPECIES: ATP-dependent Clp pro	WP_000934041.1	84 kDa		unknown	0	0	0	0	0	0	0	0	0	1	37	0	4
97	TRUE	Empty	adherence protein [Escherichia coli]	WP_001239081.1	366 kDa		unknown	0	0	0	0	35	0	0	0	0	0	0	0	0
98	TRUE	Empty	MULTISPECIES: catabolite repressor/ac	WP_000762401.1	38 kDa		unknown	0	0	0	29	0	0	1	0	4	0	0	0	0
99	TRUE	Empty	MULTISPECIES: DNA-directed RNA poly	WP_001162094.1	37 kDa		unknown	0	0	6	3	2	0	7	0	12	3	4	0	1
100	TRUE	Empty	MULTISPECIES: PTS glucose EIICB comp	WP_000475719.1	51 kDa		unknown	0	0	0	0	0	0	2	0	2	5	28	0	1
101	TRUE	Empty	ATP-dependent helicase [Escherichia c	WP_000139534.1	149 kDa		unknown	0	0	0	0	0	0	0	0	0	0	35	1	0
102	TRUE	Empty	D-erythrose-4-phosphate dehydrogena	WP_000218483.1	37 kDa		unknown	0	0	0	29	0	0	0	0	0	0	0	0	0
103	TRUE	Empty	succinate dehydrogenase flavoprotein	WP_000775544.1	64 kDa		unknown	0	0	0	0	0	0	2	0	3	7	6	0	21
104	TRUE	Empty	MULTISPECIES: paraquat-inducible pro	WP_000445547.1	60 kDa		unknown	0	0	0	0	0	0	0	0	0	36	0	0	0
105	TRUE	Empty	MULTISPECIES: murein lipoprotein [Pro	WP_000648420.1	8 kDa		unknown	0	0	0	0	0	0	7	0	6	8	14	0	0

106	TRUE	Empty	rRNA (cytidine-2'-O-)-methyltransferas	WP_000809266.1	31 kDa		unknown	0	0	2	0	0	0	0	0	33	1	0	0	0
107	TRUE	Empty	alpha-glucosidase/alpha-galactosidase	WP_000986633.1	51 kDa		unknown	0	0	0	0	0	0	8	0	4	11	3	0	6
108	TRUE	Empty	MULTISPECIES: glycine--tRNA ligase su	WP_001168544.1	35 kDa		unknown	0	0	0	34	0	0	0	0	0	0	0	0	0
109	TRUE	Empty	MULTISPECIES: DNA-binding response	WP_000611328.1	24 kDa		unknown	0	0	33	0	0	0	0	0	0	0	0	0	0
110	TRUE	Empty	ribosomal large subunit pseudouridine	WP_000846329.1	36 kDa		unknown	0	0	0	28	0	0	0	0	0	0	0	0	0
111	TRUE	Empty	MULTISPECIES: UDP-N-acetylmuramat	WP_001096048.1	54 kDa		unknown	0	0	0	0	0	0	0	0	0	36	0	0	0
112	TRUE	Empty	MULTISPECIES: 50S ribosomal protein	WP_001238917.1	15 kDa		unknown	0	3	5	1	2	0	15	0	5	2	2	0	3
113	TRUE	Empty	MULTISPECIES: DNA gyrase subunit A [	WP_001281253.1	97 kDa		unknown	0	0	0	0	0	0	0	0	0	0	20	0	10
114	TRUE	Empty	nitrate reductase subunit alpha [Esche	WP_000032958.1	141 kDa		unknown	0	0	0	0	0	0	0	0	0	0	35	0	0
115	TRUE	Empty	phosphoporin PhoE [Escherichia coli]	WP_000865540.1	41 kDa		unknown	0	0	2	7	0	0	8	0	13	3	1	0	0
116	TRUE	Empty	MULTISPECIES: ATP-dependent 6-phos	WP_001318165.1	35 kDa		unknown	0	0	0	36	0	0	0	0	0	0	0	0	0
117	TRUE	Empty	MULTISPECIES: ATP synthase subunit g	WP_000896498.1	32 kDa		unknown	0	0	17	0	0	0	1	0	13	0	0	0	0
118	TRUE	Empty	MULTISPECIES: oligopeptide transport	WP_000994905.1	37 kDa		unknown	0	0	0	32	0	0	0	0	0	0	0	0	0
119	TRUE	Empty	translocated intimin receptor Tir [Esch	WP_001339882.1	57 kDa		unknown	0	0	0	0	0	0	0	0	0	31	1	0	2
120	TRUE	Empty	MULTISPECIES: ribonucleotide monop	WP_000153129.1	27 kDa		unknown	0	0	34	0	0	0	0	0	0	0	0	0	0
121	TRUE	Empty	MULTISPECIES: ATP-dependent RNA he	WP_000219193.1	50 kDa		unknown	0	0	0	0	0	0	0	0	0	23	4	0	2
122	TRUE	Empty	chaperone protein Skp [Escherichia col	WP_000758957.1	18 kDa		unknown	0	31	0	0	0	0	0	0	0	0	0	0	0
123	TRUE	Empty	protein translocase subunit SecD [Esch	WP_000934826.1	67 kDa		unknown	0	0	0	0	0	0	0	0	0	27	6	0	0
124	TRUE	Empty	MULTISPECIES: NADH-quinone oxidore	WP_000386733.1	25 kDa		unknown	0	0	18	0	0	0	0	0	0	0	0	0	0
125	TRUE	Empty	MULTISPECIES: molecular chaperone D	WP_000516135.1	69 kDa		unknown	0	0	6	4	0	0	2	0	0	10	5	0	3
126	TRUE	Empty	phosphatidylserine decarboxylase pro	WP_000934913.1	36 kDa		unknown	0	0	0	0	0	0	26	0	0	0	0	0	0
127	TRUE	Empty	MULTISPECIES: 30S ribosomal protein	WP_000940121.1	18 kDa		unknown	0	3	3	1	2	0	6	0	10	0	3	0	0
128	TRUE	Empty	MULTISPECIES: DNA-binding response	WP_001033719.1	26 kDa		unknown	0	0	0	0	0	0	24	0	1	1	0	0	0
129	TRUE	Empty	hypothetical protein [Escherichia coli]	WP_000091467.1	27 kDa		unknown	0	0	0	0	0	0	22	0	2	0	0	0	0
130	TRUE	Empty	MULTISPECIES: purine-nucleoside phos	WP_000224879.1	26 kDa		unknown	0	0	30	0	0	0	0	0	0	0	0	0	0
131	TRUE	Empty	MULTISPECIES: valine--tRNA ligase [Es	WP_000416385.1	108 kDa		unknown	0	0	0	0	0	0	0	0	0	0	0	0	31
132	TRUE	Empty	MULTISPECIES: NAD(P)-dependent oxi	WP_000517443.1	28 kDa		unknown	0	0	1	0	0	0	0	0	29	0	0	0	0
133	TRUE	Empty	MULTISPECIES: ADP-L-glycero-D-mann	WP_000587764.1	35 kDa		unknown	0	0	0	28	0	0	0	0	0	0	0	0	0
134	TRUE	Empty	transcriptional regulator [Escherichia c	WP_000836620.1	23 kDa		unknown	0	0	25	0	0	0	0	0	0	0	0	0	0
135	TRUE	Empty	DUF3971 domain-containing protein [E	WP_001253536.1	139 kDa		unknown	0	0	0	0	0	0	0	0	0	0	31	0	0
136	TRUE	Empty	MULTISPECIES: N utilization substance	WP_000801125.1	16 kDa		unknown	0	30	0	0	0	0	0	0	0	0	0	0	0
137	TRUE	Empty	MULTISPECIES: methionine ABC transp	WP_000874226.1	29 kDa		unknown	0	0	1	0	0	0	24	0	0	0	0	0	0
138	TRUE	Empty	type I restriction-modification system	WP_000202819.1	58 kDa		unknown	0	0	0	0	0	0	0	0	0	28	0	0	0
139	TRUE	Empty	phospholipid ABC transporter ATP-bin	WP_000438249.1	29 kDa		unknown	0	0	2	0	0	0	0	0	20	0	0	0	0
140	TRUE	Empty	MULTISPECIES: ATP-dependent zinc m	WP_001107467.1	71 kDa		unknown	0	0	0	0	0	0	2	0	0	26	0	0	0
141	TRUE	Empty	MULTISPECIES: HslU--HslV peptidase A	WP_001293344.1	50 kDa		unknown	0	0	4	2	2	0	9	0	0	10	0	0	0
142	TRUE	Empty	MULTISPECIES: arginine ABC transport	WP_000027205.1	27 kDa		unknown	0	0	0	0	0	0	22	0	2	0	0	0	0
143	TRUE	Empty	MULTISPECIES: universal stress protein	WP_000323555.1	16 kDa		unknown	0	24	0	0	0	0	0	0	0	0	0	0	0
144	TRUE	Empty	MULTISPECIES: transcription terminati	WP_001054527.1	47 kDa		unknown	0	0	1	9	0	0	3	0	1	8	0	0	0
145	TRUE	Empty	MULTISPECIES: chaperone protein Clp	WP_001235102.1	96 kDa		unknown	0	0	0	1	0	0	0	0	0	4	7	0	12
146	TRUE	Empty	DNA gyrase subunit B [Escherichia coli]	WP_000072051.1	90 kDa		unknown	0	0	0	1	1	0	0	0	0	1	15	0	3
147	TRUE	Empty	MULTISPECIES: hypothetical protein [E	WP_000228584.1	16 kDa		unknown	0	19	0	0	0	0	0	0	0	0	0	0	0
148	TRUE	Empty	MULTISPECIES: 50S ribosomal protein	WP_000424395.1	22 kDa		unknown	0	9	1	0	0	0	4	0	3	0	0	0	0
149	TRUE	Empty	MULTISPECIES: cytochrome bd-I ubiqu	WP_000568275.1	42 kDa		unknown	0	0	0	0	0	0	0	0	6	0	10	0	0
150	TRUE	Empty	Zn-dependent oxidoreductase [Escheri	WP_000836074.1	36 kDa		unknown	0	0	0	23	0	0	0	0	0	0	0	0	0
151	TRUE	Empty	MULTISPECIES: 50S ribosomal protein	WP_001207201.1	18 kDa		unknown	0	20	0	0	0	0	0	0	0	0	0	0	0
152	TRUE	Empty	translocation/assembly module TamB	WP_000060877.1	137 kDa		unknown	0	0	0	0	0	0	0	0	0	0	24	1	0
153	TRUE	Empty	beta-galactosidase [Escherichia coli]	WP_000177864.1	116 kDa		unknown	0	0	0	0	2	0	1	0	1	6	4	0	5
154	TRUE	Empty	bifunctional UDP-glucuronic acid oxida	WP_000860292.1	74 kDa		unknown	0	0	0	0	1	0	0	0	0	0	2	0	22
155	TRUE	Empty	MULTISPECIES: biotin synthase [Proteo	WP_000951213.1	39 kDa		unknown	0	0	0	23	0	0	0	0	0	0	0	0	0
156	TRUE	Empty	MULTISPECIES: 50S ribosomal protein	WP_001096680.1	25 kDa		unknown	0	0	6	0	0	0	8	0	2	2	0	0	2
157	TRUE	Empty	MULTISPECIES: fumarate reductase fla	WP_001192980.1	66 kDa		unknown	0	0	0	0	0	0	0	0	1	11	4	0	10
158	TRUE	Empty	MULTISPECIES: polyphosphate kinase [	WP_001296288.1	80 kDa		unknown	0	0	0	0	0	0	1	0	1	0	6	0	15
159	TRUE	Empty	MULTISPECIES: protein translocase sub	WP_000046637.1	35 kDa		unknown	0	0	2	0	2	0	10	0	3	2	3	0	2
160	TRUE	Empty	enterobactin synthase subunit F [Esche	WP_000077760.1	142 kDa		unknown	0	0	0	0	0	0	0	0	0	0	23	0	0
161	TRUE	Empty	MULTISPECIES: 50S ribosomal protein	WP_000091955.1	9 kDa		unknown	0	12	2	0	3	0	1	0	0	0	0	0	0
162	TRUE	Empty	MULTISPECIES: ATP-dependent Clp pro	WP_000130305.1	46 kDa		unknown	0	0	2	14	0	0	3	0	0	1	1	0	0
163	TRUE	Empty	LPS-assembly protein LptD [Escherichia	WP_000746165.1	90 kDa		unknown	0	0	0	0	0	0	0	0	0	0	18	0	0
164	TRUE	Empty	3-methyl-2-oxobutanoate hydroxymet	WP_000805464.1	28 kDa		unknown	0	0	0	0	0	0	14	0	0	1	0	0	0
165	TRUE	Empty	MULTISPECIES: RNA polymerase-assoc	WP_001117001.1	110 kDa		unknown	0	0	0	0	2	0	0	0	0	0	4	0	11
166	TRUE	Empty	MULTISPECIES: 50S ribosomal protein	WP_001216368.1	14 kDa		unknown	0	16	0	0	0	0	4	0	2	0	0	0	0
167	TRUE	Empty	MULTISPECIES: guanosine monophosp	WP_001217338.1	37 kDa		unknown	0	0	0	20	0	0	0	0	0	0	0	0	0
168	TRUE	Empty	MULTISPECIES: condensin subunit E [E	WP_001295931.1	27 kDa		unknown	0	0	0	0	0	0	20	0	0	0	0	0	0
169	TRUE	Empty	MULTISPECIES: dihydroorotate dehydr	WP_001295934.1	37 kDa		unknown	0	0	0	25	0	0	0	0	0	0	0	0	0
170	TRUE	Empty	MULTISPECIES: protein-export protein	WP_000003382.1	17 kDa		unknown	0	22	0	0	0	0	0	0	0	0	0	0	0
171	TRUE	Empty	MULTISPECIES: 50S ribosomal protein	WP_000091945.1	19 kDa		unknown	0	0	12	0	0	0	6	0	2	0	0	0	0
172	TRUE	Empty	MULTISPECIES: glycosyl transferase [Es	WP_000799972.1	38 kDa		unknown	0	0	0	18	0	0	0	0	0	2	0	0	0
173	TRUE	Empty	peptidase S8 [Escherichia coli]	WP_000970615.1	82 kDa		unknown	0	0	4	0	2	0	5	0	0	0	11	0	0
174	TRUE	Empty	MULTISPECIES: DNA-binding transcript	WP_001262188.1	30 kDa		unknown	0	0	0	0	0	0	0	0	24	0	0	0	0
175	TRUE	Empty	MULTISPECIES: NAD(P)(+) transhydrog	WP_001298661.1	55 kDa		unknown	0	0	0	0	0	0	0	0	0	9	10	0	0
176	TRUE	Empty	two-component system sensor histidin	WP_012578962.1	135 kDa		unknown	0	0	0	0	0	0	0	0	0	0	22	0	0
177	TRUE	Empty	carboxy-S-adenosyl-L-methionine synt	WP_000019589.1	28 kDa		unknown	0	0	24	0	0	0	0	0	0	0	0	0	0
178	TRUE	Empty	MULTISPECIES: CesD/SycD/LcrH family	WP_000087467.1	18 kDa		unknown	0	21	0	0	0	0	0	0	0	0	0	0	0
179	TRUE	Empty	MULTISPECIES: ribonuclease E/G [Prot	WP_000123197.1	55 kDa		unknown	0	0	0	0	0	0	0	0	0	22	0	0	0

180	TRUE	Empty	MULTISPECIES: fatty acid metabolism t	WP_000234823.1	27 kDa		unknown	0	0	23	0	0	0	0	0	0	0	0	0	0
181	TRUE	Empty	MULTISPECIES: cell division protein Fts	WP_000462776.1	40 kDa		unknown	0	0	1	10	0	0	4	0	2	2	0	0	0
182	TRUE	Empty	MULTISPECIES: transcriptional regulato	WP_000611911.1	28 kDa		unknown	0	0	1	0	0	0	18	0	0	0	0	0	0
183	TRUE	Empty	MULTISPECIES: Appr-1-p processing pr	WP_000634203.1	41 kDa		unknown	0	0	0	12	0	0	0	0	0	0	0	0	0
184	TRUE	Empty	ADP-heptose--LPS heptosyltransferase	WP_000699241.1	39 kDa		unknown	0	0	0	20	0	0	0	0	0	0	0	0	0
185	TRUE	Empty	MULTISPECIES: nucleoside-diphosphat	WP_000963849.1	15 kDa		unknown	0	22	0	0	0	0	0	0	0	0	0	0	0
186	TRUE	Empty	MULTISPECIES: 4-hydroxy-3-methylbut	WP_001166403.1	35 kDa		unknown	0	0	0	19	0	0	0	0	0	0	0	0	0
187	TRUE	Empty	phospholipase [Escherichia coli]	WP_000102671.1	38 kDa		unknown	0	0	0	23	0	0	0	0	0	0	0	0	0
188	TRUE	Empty	MULTISPECIES: AsnC family transcripti	WP_000432970.1	17 kDa		unknown	0	20	0	0	0	0	0	0	0	0	0	0	0
189	TRUE	Empty	MULTISPECIES: translational GTPase Ty	WP_000570668.1	67 kDa		unknown	0	0	0	0	3	0	0	0	0	0	17	0	2
190	TRUE	Empty	MULTISPECIES: acyl-CoA thioester hyd	WP_001098384.1	16 kDa		unknown	0	17	0	0	0	0	0	0	0	0	0	0	0
191	TRUE	Empty	MULTISPECIES: 23S rRNA pseudouridyl	WP_001291206.1	33 kDa		unknown	0	0	0	0	0	0	18	0	2	0	0	0	0
192	TRUE	Empty	MULTISPECIES: nucleoside-specific cha	WP_001295328.1	34 kDa		unknown	0	0	0	0	0	0	18	0	0	0	0	0	0
193	TRUE	Empty	MULTISPECIES: outer membrane prote	WP_001296286.1	37 kDa		unknown	0	0	0	19	0	0	0	0	0	0	0	0	0
194	TRUE	Empty	alanine--tRNA ligase [Escherichia coli]	WP_000047168.1	96 kDa		unknown	0	0	1	1	0	0	0	0	0	0	6	0	7
195	TRUE	Empty	MULTISPECIES: oligopeptide ABC trans	WP_000110950.1	37 kDa		unknown	0	0	0	21	0	0	0	0	0	0	0	0	0
196	TRUE	Empty	heavy metal-responsive transcriptional	WP_000285619.1	16 kDa		unknown	0	20	0	0	0	0	0	0	0	0	0	0	0
197	TRUE	Empty	lipid ABC transporter permease/ATP-b	WP_000551260.1	65 kDa		unknown	0	0	0	0	0	0	0	0	0	19	2	0	0
198	TRUE	Empty	protein translocase subunit SecA [Esch	WP_000905766.1	102 kDa		unknown	0	0	0	0	0	0	0	0	0	0	6	0	10
199	TRUE	Empty	MULTISPECIES: enolase [Proteobacteri	WP_000036723.1	46 kDa		unknown	0	0	0	0	0	0	7	0	5	3	3	0	1
200	TRUE	Empty	MULTISPECIES: IclR family transcriptior	WP_000514241.1	31 kDa		unknown	0	0	0	0	0	0	0	0	17	0	0	0	0
201	TRUE	Empty	4-hydroxy-tetrahydrodipicolinate redu	WP_000543583.1	29 kDa		unknown	0	0	0	0	0	0	15	0	0	0	0	0	0
202	TRUE	Empty	MULTISPECIES: 5'-methylthioadenosin	WP_000689844.1	24 kDa		unknown	0	0	20	0	0	0	0	0	0	0	0	0	0
203	TRUE	Empty	MULTISPECIES: molecular chaperone D	WP_001118464.1	41 kDa		unknown	0	0	8	10	0	0	0	0	0	0	0	0	0
204	TRUE	Empty	MULTISPECIES: GDP-mannose 4,6-deh	WP_001140914.1 (+1	42 kDa		unknown	0	0	7	6	5	0	0	0	0	0	0	0	0
205	TRUE	Empty	tRNA 2-thiocytidine(32) synthetase Ttc	WP_001157409.1	36 kDa		unknown	0	0	0	17	0	0	0	0	0	0	0	0	0
206	TRUE	Empty	2-deoxy-D-gluconate 3-dehydrogenase	WP_000603516.1	27 kDa		unknown	0	0	18	0	0	0	0	0	0	0	0	0	0
207	TRUE	Empty	MULTISPECIES: DeoR/GlpR transcriptio	WP_000679501.1	28 kDa		unknown	0	0	0	0	0	0	12	0	0	0	0	0	0
208	TRUE	Empty	7-cyano-7-deazaguanine synthase Que	WP_000817225.1	25 kDa		unknown	0	0	17	0	0	0	0	0	0	0	0	0	0
209	TRUE	Empty	asparagine synthetase A [Escherichia c	WP_000845130.1	37 kDa		unknown	0	0	2	2	0	0	5	0	8	1	0	0	0
210	TRUE	Empty	MULTISPECIES: elongation factor P--(R	WP_000004771.1	37 kDa		unknown	0	0	0	16	0	0	0	0	0	0	0	0	0
211	TRUE	Empty	glycerol-3-phosphate dehydrogenase [	WP_000448158.1	57 kDa		unknown	0	0	0	0	0	0	0	0	0	17	0	0	0
212	TRUE	Empty	FAD-binding oxidoreductase [Escherich	WP_000612987.1	113 kDa		unknown	0	0	0	0	0	0	0	0	0	0	4	0	11
213	TRUE	Empty	endonuclease VIII [Escherichia coli]	WP_001114005.1	30 kDa		unknown	0	0	0	0	0	0	15	0	0	0	0	0	0
214	TRUE	Empty	MULTISPECIES: ribose-phosphate pyro	WP_001298109.1	34 kDa		unknown	0	4	9	2	3	0	0	0	0	0	0	0	0
215	TRUE	Empty	MULTISPECIES: serine/threonine trans	WP_000450473.1	47 kDa		unknown	0	0	0	4	5	0	0	0	0	0	4	0	2
216	TRUE	Empty	bifunctional aspartokinase II/homoseri	WP_000110759.1	89 kDa		unknown	0	0	0	0	0	0	0	0	0	0	17	0	0
217	TRUE	Empty	MULTISPECIES: GTP-binding protein [P	WP_000183349.1	24 kDa		unknown	0	0	15	0	0	0	0	0	0	0	0	0	0
218	TRUE	Empty	MULTISPECIES: universal stress protein	WP_000323571.1	16 kDa		unknown	0	12	0	0	0	0	0	0	0	0	0	0	0
219	TRUE	Empty	glutamate racemase [Escherichia coli]	WP_000959841.1 (+1	29 kDa		unknown	0	0	1	0	0	0	0	0	16	0	0	0	0
220	TRUE	Empty	MULTISPECIES: 23S rRNA (guanosine(2	WP_001293282.1	27 kDa		unknown	0	0	16	0	0	0	0	0	0	0	0	0	0
221	TRUE	Empty	MULTISPECIES: stringent starvation pro	WP_000257293.1	24 kDa		unknown	0	0	13	0	0	0	0	0	0	0	0	0	0
222	TRUE	Empty	MULTISPECIES: 50S ribosomal protein	WP_000271401.1	12 kDa		unknown	0	12	0	0	0	0	0	0	0	0	0	0	0
223	TRUE	Empty	MULTISPECIES: tRNA 4-thiouridine(8) s	WP_000668665.1	55 kDa		unknown	0	0	0	0	0	0	0	0	0	10	0	0	0
224	TRUE	Empty	MULTISPECIES: elongation factor 4 [En	WP_000790169.1	67 kDa		unknown	0	0	0	2	5	0	0	0	0	0	2	0	6
225	TRUE	Empty	MULTISPECIES: PTS fructose transporte	WP_000854450.1	58 kDa		unknown	0	0	0	0	0	0	0	0	0	11	5	0	0
226	TRUE	Empty	MULTISPECIES: methyltransferase [Ent	WP_000997418.1	38 kDa		unknown	0	0	0	12	0	0	1	0	0	0	0	0	0
227	TRUE	Empty	MULTISPECIES: PTS N-acetyl glucosami	WP_001023115.1	68 kDa		unknown	0	0	0	0	0	0	0	0	0	2	13	0	0
228	TRUE	Empty	transcriptional regulator ExuR [Escheri	WP_000406486.1	30 kDa		unknown	0	0	0	0	0	0	0	0	12	0	0	0	0
229	TRUE	Empty	hypothetical protein [Escherichia coli]	WP_000544657.1	90 kDa		unknown	0	0	0	0	2	0	0	0	3	5	0	0	0
230	TRUE	Empty	MULTISPECIES: formyltetrahydrofolate	WP_000555849.1	32 kDa		unknown	0	0	0	0	0	0	0	0	7	0	0	0	0
231	TRUE	Empty	LPS core heptose(II) kinase RfaY [Esche	WP_000615256.1	27 kDa		unknown	0	0	12	0	0	0	0	0	0	0	0	0	0
232	TRUE	Empty	MULTISPECIES: autonomous glycyl radi	WP_000627804.1	14 kDa	TRUE	unknown	0	9	0	0	0	0	0	0	0	0	0	0	0
233	TRUE	Empty	MULTISPECIES: ribosomal RNA small su	WP_000932839.1	23 kDa		unknown	0	0	12	0	0	0	0	0	0	0	0	0	0
234	TRUE	Empty	lipoprotein-releasing system ATP-bindi	WP_001033687.1	26 kDa		unknown	0	0	12	0	0	0	0	0	0	0	0	0	0
235	TRUE	Empty	MULTISPECIES: S-adenosylmethionine	WP_001062128.1	42 kDa		unknown	0	0	1	0	1	0	2	0	0	8	0	0	2
236	TRUE	Empty	MULTISPECIES: ferrochelatase [Entero	WP_001250115.1	36 kDa		unknown	0	0	0	13	0	0	0	0	0	0	0	0	0
237	TRUE	Empty	MULTISPECIES: cyclic di-GMP phospho	WP_001295808.1	41 kDa		unknown	0	0	0	11	0	0	0	0	0	0	0	0	0
238	TRUE	Empty	MULTISPECIES: CDP-diacylglycerol pyro	WP_001298413.1	28 kDa		unknown	0	0	0	0	0	0	0	0	15	0	0	0	0
239	TRUE	Empty	MULTISPECIES: 50S ribosomal protein	WP_001096200.1	20 kDa		unknown	0	2	7	0	0	0	3	0	2	0	0	0	0
240	TRUE	Empty	MULTISPECIES: 50S ribosomal protein	WP_000065253.1	13 kDa		unknown	0	9	0	0	0	0	0	0	0	0	3	0	0
241	TRUE	Empty	MULTISPECIES: lipoprotein NlpA [Enter	WP_000779411.1	29 kDa		unknown	0	0	0	0	0	0	12	0	0	0	0	0	0
242	TRUE	Empty	MULTISPECIES: phosphoenolpyruvate	WP_001005586.1	99 kDa		unknown	0	0	0	0	1	0	0	0	0	0	8	0	2
243	TRUE	Empty	MULTISPECIES: protein translocase sub	WP_001118861.1	49 kDa		unknown	0	0	0	2	0	0	0	0	0	0	6	0	0
244	TRUE	Empty	bifunctional phosphoribosylaminoimid	WP_001187528.1	57 kDa		unknown	0	0	0	0	0	0	0	0	0	14	0	0	0
245	TRUE	Empty	MULTISPECIES: glycogen synthase [Pro	WP_001197646.1	53 kDa		unknown	0	0	0	0	0	0	1	0	0	11	1	0	1
246	TRUE	Empty	cellulose biosynthesis protein BcsE [Es	WP_001204947.1 (+1	59 kDa		unknown	0	0	0	0	0	0	0	0	0	10	0	0	0
247	TRUE	Empty	tRNA/rRNA methyltransferase [Escheri	WP_001223158.1	25 kDa		unknown	0	0	9	0	0	0	0	0	0	0	0	0	0
248	TRUE	Empty	MULTISPECIES: succinate dehydrogena	WP_001235263.1	27 kDa		unknown	0	0	12	0	0	0	0	0	0	0	0	0	0
249	TRUE	Empty	aconitate hydratase B [Escherichia coli	WP_012578834.1	94 kDa		unknown	0	0	0	0	2	0	0	0	0	0	2	0	7
250	TRUE	Empty	N-acetylmannosamine-6-phosphate 2-	WP_012579008.1	24 kDa		unknown	0	0	13	0	0	0	0	0	0	0	0	0	0
251	TRUE	Empty	MULTISPECIES: translation initiation fa	WP_001700733.1	21 kDa		unknown	0	4	5	0	0	0	1	0	1	0	0	0	0
252	TRUE	Empty	MULTISPECIES: septum site-determinir	WP_000072536.1	25 kDa		unknown	0	0	9	0	0	0	0	0	0	0	0	0	0
253	TRUE	Empty	Cu+ exporting ATPase [Escherichia coli	WP_000083997.1	88 kDa		unknown	0	0	0	0	0	0	0	0	0	0	5	0	0

254	TRUE	Empty	MULTISPECIES: galactitol utilization op	WP_000178556.1 (+1	28 kDa		unknown	0	0	0	0	0	0	0	0	13	0	0	0	0
255	TRUE	Empty	MULTISPECIES: outer membrane prote	WP_000197686.1	28 kDa		unknown	0	0	7	0	0	0	0	0	0	0	0	0	0
256	TRUE	Empty	MULTISPECIES: phosphoenolpyruvate s	WP_000368046.1	31 kDa		unknown	0	0	0	0	0	0	9	0	0	0	0	0	0
257	TRUE	Empty	MULTISPECIES: GntR family transcripti	WP_000434049.1	28 kDa		unknown	0	0	0	0	0	0	10	0	0	0	0	0	0
258	TRUE	Empty	MULTISPECIES: acyl-[acyl-carrier-prote	WP_000565966.1	28 kDa		unknown	0	0	0	0	0	0	8	0	0	0	0	0	0
259	TRUE	Empty	MULTISPECIES: glutamine ABC transpo	WP_000569080.1	27 kDa		unknown	0	0	10	0	0	0	0	0	0	0	0	0	0
260	TRUE	Empty	MULTISPECIES: fumarate reductase iro	WP_000829498.1	27 kDa		unknown	0	0	13	0	0	0	0	0	0	0	0	0	0
261	TRUE	Empty	MULTISPECIES: phosphoserine phosph	WP_001132956.1	35 kDa		unknown	0	0	0	7	0	0	0	0	0	6	0	0	0
262	TRUE	Empty	sugar phosphatase [Escherichia coli]	WP_001203417.1	23 kDa		unknown	0	0	11	0	0	0	0	0	0	0	0	0	0
263	TRUE	Empty	MULTISPECIES: lactoylglutathione lyase	WP_001237796.1	15 kDa		unknown	0	6	0	0	0	0	0	0	0	0	0	0	0
264	TRUE	Empty	DNA polymerase III subunit alpha [Esch	WP_001294779.1	130 kDa		unknown	0	0	0	0	0	0	0	0	0	0	10	0	0
265	TRUE	Empty	GDP-fucose synthetase [Escherichia co	WP_000089908.1	36 kDa		unknown	0	0	3	6	0	0	0	0	0	0	0	0	0
266	TRUE	Empty	UDP-3-O-(3-hydroxymyristoyl)glucosar	WP_000095071.1	36 kDa		unknown	0	0	0	12	0	0	0	0	0	0	0	0	0
267	TRUE	Empty	MULTISPECIES: 3-dehydroquinate synt	WP_000439850.1	39 kDa		unknown	0	0	0	10	0	0	0	0	0	0	0	0	0
268	TRUE	Empty	pyruvate:ferredoxin (flavodoxin) oxido	WP_000628227.1	129 kDa		unknown	0	0	0	0	0	0	0	0	0	0	6	0	0
269	TRUE	Empty	ethanolamine utilization protein EutQ	WP_000733877.1	25 kDa		unknown	0	0	0	0	0	0	10	0	0	0	0	0	0
270	TRUE	Empty	DUF484 family protein [Escherichia col	WP_000812801.1	27 kDa		unknown	0	0	11	0	0	0	0	0	0	0	0	0	0
271	TRUE	Empty	MULTISPECIES: GTP cyclohydrolase I Fe	WP_001139613.1	25 kDa		unknown	0	0	11	0	0	0	0	0	0	0	0	0	0
272	TRUE	Empty	isoleucine--tRNA ligase [Escherichia co	WP_001286824.1	104 kDa		unknown	0	0	0	0	0	0	0	0	0	0	0	0	12
273	TRUE	Empty	MULTISPECIES: uridine kinase [Proteob	WP_001295424.1	24 kDa		unknown	0	0	12	0	0	0	0	0	0	0	0	0	0
274	TRUE	Empty	MULTISPECIES: DNA topoisomerase I [E	WP_001295576.1	97 kDa		unknown	0	0	0	0	0	0	0	0	0	0	2	0	7
275	TRUE	Empty	MULTISPECIES: IMP dehydrogenase [Er	WP_001296289.1	52 kDa		unknown	0	0	0	1	0	0	0	0	0	10	0	0	0
276	TRUE	Empty	MULTISPECIES: response regulator [Pro	WP_000126500.1	25 kDa		unknown	0	0	11	0	0	0	0	0	0	0	0	0	0
277	TRUE	Empty	transporter [Escherichia coli]	WP_003370406.1	25 kDa		unknown	0	0	9	0	0	0	0	0	0	0	0	0	0
278	TRUE	Empty	chromosome partition protein MukB [E	WP_000572652.1	170 kDa		unknown	0	0	0	0	0	0	0	0	0	0	10	0	0
279	TRUE	Empty	MULTISPECIES: Holliday junction branc	WP_000580323.1	22 kDa		unknown	0	0	10	0	0	0	0	0	0	0	0	0	0
280	TRUE	Empty	MULTISPECIES: hypothetical protein [E	WP_000609744.1	26 kDa		unknown	0	0	10	0	0	0	0	0	0	0	0	0	0
281	TRUE	Empty	MULTISPECIES: L-threonine 3-dehydrog	WP_000646018.1	37 kDa		unknown	0	0	0	7	0	0	0	0	0	0	0	0	0
282	TRUE	Empty	peptidylprolyl isomerase [Escherichia c	WP_000969352.1	68 kDa		unknown	0	0	0	0	0	0	0	0	0	10	0	0	0
283	TRUE	Empty	phospho-2-dehydro-3-deoxyheptonate	WP_001109184.1	38 kDa		unknown	0	0	0	10	0	0	0	0	0	0	0	0	0
284	TRUE	Empty	MULTISPECIES: pyridoxine 5'-phosphat	WP_001309669.1	26 kDa		unknown	0	0	0	0	0	0	9	0	0	0	0	0	0
285	TRUE	Empty	MULTISPECIES: transketolase [Proteob	WP_000098614.1	72 kDa		unknown	0	0	0	0	0	0	0	0	0	0	2	0	3
286	TRUE	Empty	acetolactate synthase [Escherichia coli	WP_000168501.1	60 kDa		unknown	0	0	0	0	0	0	0	0	0	10	0	0	0
287	TRUE	Empty	MULTISPECIES: cell division ATP-bindin	WP_000617723.1	24 kDa		unknown	0	0	8	0	0	0	0	0	0	0	0	0	0
288	TRUE	Empty	hypothetical protein [Escherichia coli]	WP_000736352.1	181 kDa		unknown	0	0	0	0	0	0	0	0	0	0	0	0	8
289	TRUE	Empty	MULTISPECIES: DNA replication protei	WP_000799911.1	28 kDa		unknown	0	0	0	0	0	0	0	0	8	0	0	0	0
290	TRUE	Empty	MULTISPECIES: peptidyl-prolyl cis-tran	WP_000838264.1	29 kDa		unknown	0	0	0	0	0	0	0	0	8	0	0	0	0
291	TRUE	Empty	bifunctional 3-demethylubiquinone 3-(	WP_000990770.1	27 kDa		unknown	0	0	0	0	0	0	6	0	0	0	0	0	0
292	TRUE	Empty	protein EaeB [Escherichia coli]	WP_001091991.1	33 kDa		unknown	0	0	0	0	0	0	3	0	5	1	0	0	0
293	TRUE	Empty	ribosomal RNA small subunit methyltra	WP_012578994.1	27 kDa		unknown	0	0	6	0	0	0	4	0	0	0	0	0	0
294	TRUE	Empty	MULTISPECIES: acetyl-CoA carboxylase	WP_000055746.1	35 kDa		unknown	0	0	1	0	0	0	1	0	3	0	0	0	0
295	TRUE	Empty	MULTISPECIES: cytochrome ubiquinol o	WP_000467180.1	74 kDa		unknown	0	0	0	0	0	0	0	0	0	2	5	0	1
296	TRUE	Empty	MULTISPECIES: 50S ribosomal protein l	WP_000941212.1	15 kDa		unknown	0	3	2	0	0	0	3	0	2	0	0	0	0
297	TRUE	Empty	MULTISPECIES: SlyA family transcriptio	WP_000445640.1	16 kDa		unknown	0	9	0	0	0	0	0	0	0	0	0	0	0
298	TRUE	Empty	MULTISPECIES: enoyl-ACP reductase [E	WP_000506492.1	28 kDa		unknown	0	0	0	0	0	0	1	0	7	0	0	0	0
299	TRUE	Empty	MULTISPECIES: lipoprotein NlpI [Protec	WP_000802080.1	34 kDa		unknown	0	0	0	0	0	0	0	0	7	0	0	0	0
300	TRUE	Empty	MULTISPECIES: maltoporin [Escherichia	WP_000973666.1	50 kDa		unknown	0	0	0	0	0	0	0	0	0	0	5	0	0
301	TRUE	Empty	MULTISPECIES: ribosomal RNA small su	WP_001065363.1	30 kDa		unknown	0	0	0	0	0	0	0	0	6	0	0	0	0
302	TRUE	Empty	DNA helicase IV [Escherichia coli]	WP_001340087.1	78 kDa		unknown	0	0	0	0	0	0	0	0	0	0	6	0	0
303	TRUE	Empty	O-acetyltransferase [Escherichia coli]	WP_012578939.1	25 kDa		unknown	0	0	9	0	0	0	0	0	0	0	0	0	0
304	TRUE	Empty	MULTISPECIES: nucleoside triphosphat	WP_001071648.1	30 kDa		unknown	0	0	0	0	0	0	7	0	0	0	0	0	0
305	TRUE	Empty	DeoR/GlpR transcriptional regulator [E	WP_000022208.1	29 kDa		unknown	0	0	0	0	0	0	4	0	0	0	0	0	0
306	TRUE	Empty	MULTISPECIES: energy-dependent tran	WP_000046749.1	62 kDa		unknown	0	0	0	2	0	0	0	0	0	5	1	0	0
307	TRUE	Empty	MULTISPECIES: hypothetical protein [P	WP_000272188.1	15 kDa		unknown	0	7	0	0	0	0	0	0	0	0	0	0	0
308	TRUE	Empty	MULTISPECIES: aspartate 1-decarboxyl	WP_000621515.1	14 kDa		unknown	0	8	0	0	0	0	0	0	0	0	0	0	0
309	TRUE	Empty	transcriptional regulator [Escherichia c	WP_000644913.1	38 kDa		unknown	0	0	0	6	0	0	0	0	0	0	0	0	0
310	TRUE	Empty	MULTISPECIES: ADP-ribose pyrophosph	WP_000917117.1	24 kDa		unknown	0	0	7	0	0	0	0	0	0	0	0	0	0
311	TRUE	Empty	MULTISPECIES: formamidopyrimidine-[	WP_001114533.1	30 kDa		unknown	0	0	0	0	0	0	0	0	7	0	0	0	0
312	TRUE	Empty	HNH endonuclease [Escherichia coli]	WP_001124204.1	24 kDa		unknown	0	0	6	0	0	0	0	0	0	0	0	0	0
313	TRUE	Empty	MULTISPECIES: class II fructose-bispho	WP_000034375.1	39 kDa		unknown	0	0	0	4	0	0	0	0	1	0	0	0	0
314	TRUE	Empty	MULTISPECIES: alkyl hydroperoxide red	WP_000052796.1	21 kDa		unknown	0	3	2	0	0	0	0	0	1	0	0	0	0
315	TRUE	Empty	bifunctional imidazole glycerol-phosph	WP_000080126.1	40 kDa		unknown	0	0	3	0	0	0	2	0	2	0	0	0	0
316	TRUE	Empty	3-deoxy-manno-octulosonate cytidylyl	WP_000011614.1	28 kDa		unknown	0	0	1	0	0	0	5	0	0	0	0	0	0
317	TRUE	Empty	MULTISPECIES: uroporphyrinogen deca	WP_000137653.1	39 kDa		unknown	0	0	0	5	0	0	0	0	0	0	0	0	0
318	TRUE	Empty	MipA/OmpV family protein [Escherichi	WP_000163767.1	28 kDa		unknown	0	0	7	0	0	0	0	0	0	0	0	0	0
319	TRUE	Empty	MULTISPECIES: IclR family transcriptior	WP_000226409.1	30 kDa		unknown	0	0	0	0	0	0	6	0	1	0	0	0	0
320	TRUE	Empty	6-phosphofructokinase II [Escherichia c	WP_000251756.1	33 kDa		unknown	0	0	0	6	0	0	1	0	0	0	0	0	0
321	TRUE	Empty	MULTISPECIES: Fe-S biogenesis protein	WP_000619389.1	21 kDa		unknown	0	0	6	0	0	0	0	0	0	0	0	0	0
322	TRUE	Empty	MULTISPECIES: outer membrane chann	WP_000735289.1	54 kDa		unknown	0	0	0	0	0	0	0	0	0	5	0	0	0
323	TRUE	Empty	type III secretion system protein SepQ	WP_000803678.1	35 kDa		unknown	0	0	0	0	0	0	0	0	6	0	0	0	0
324	TRUE	Empty	PKHD-type hydroxylase [Escherichia co	WP_000990166.1	25 kDa		unknown	0	0	6	0	0	0	0	0	0	0	0	0	0
325	TRUE	Empty	acetolactate synthase 2 catalytic subur	WP_001012591.1	59 kDa		unknown	0	0	0	0	0	0	0	0	0	7	0	0	0
326	TRUE	Empty	exodeoxyribonuclease V subunit beta [	WP_001386407.1	134 kDa		unknown	0	0	0	0	0	0	0	0	0	0	7	0	0
327	TRUE	Empty	cell division protein DamX [Escherichia	WP_000343196.1	46 kDa		unknown	0	0	0	0	0	0	0	0	0	4	0	0	0

328	TRUE	Empty	hypothetical protein [Escherichia coli]	WP_000013660.1	13 kDa		unknown	0	4	0	0	1	0	2	0	0	0	0	0	0
329	TRUE	Empty	MULTISPECIES: threonine--tRNA ligase	WP_001144202.1	74 kDa		unknown	0	0	0	0	3	0	0	0	0	0	2	0	2
330	TRUE	Empty	NAD(P)(+) transhydrogenase (Re/Si-spe	WP_000014031.1	49 kDa		unknown	0	0	0	0	0	0	0	0	0	1	4	0	0
331	TRUE	Empty	MULTISPECIES: phosphoenolpyruvate s	WP_000069375.1	87 kDa		unknown	0	0	0	0	0	0	0	0	0	0	4	1	0
332	TRUE	Empty	flagellar hook-associated protein FlgK [	WP_000096508.1	58 kDa		unknown	0	0	0	0	0	0	0	0	0	6	0	0	0
333	TRUE	Empty	Repressor protein CI [Escherichia coli]	WP_000107166.1	35 kDa		unknown	0	0	0	6	0	0	0	0	0	0	0	0	0
334	TRUE	Empty	MULTISPECIES: transporter [Enterobac	WP_000743444.1 (+1	36 kDa		unknown	0	0	0	5	0	0	0	0	0	0	0	0	0
335	TRUE	Empty	MULTISPECIES: L-fucose mutarotase [P	WP_000920840.1	15 kDa		unknown	0	5	0	0	0	0	0	0	0	0	0	0	0
336	TRUE	Empty	MULTISPECIES: DNA-binding protein [E	WP_000988707.1	16 kDa		unknown	0	6	0	0	0	0	0	0	0	0	0	0	0
337	TRUE	Empty	ribonucleoside-diphosphate reductase	WP_001075184.1	86 kDa		unknown	0	0	0	0	0	0	0	0	0	0	5	0	0
338	TRUE	Empty	MULTISPECIES: 30S ribosomal protein S	WP_001118930.1	12 kDa		unknown	0	4	0	0	0	0	0	0	0	0	0	0	0
339	TRUE	Empty	MULTISPECIES: redox-regulated molec	WP_001135574.1	33 kDa		unknown	0	0	0	6	0	0	0	0	0	0	0	0	0
340	TRUE	Empty	MULTISPECIES: DNA-binding response	WP_001157751.1	27 kDa		unknown	0	0	0	0	0	0	6	0	0	0	0	0	0
341	TRUE	Empty	triose-phosphate isomerase [Escherich	WP_001216322.1	27 kDa		unknown	0	0	5	0	0	0	0	0	0	0	0	0	0
342	TRUE	Empty	DNA-binding response regulator [Esche	WP_000070494.1	24 kDa		unknown	0	0	5	0	0	0	0	0	0	0	0	0	0
343	TRUE	Empty	acetate kinase [Escherichia coli]	WP_000095708.1	43 kDa		unknown	0	0	0	3	0	0	0	0	0	0	0	0	0
344	TRUE	Empty	MULTISPECIES: aminocyclopropane-1-c	WP_001128237.1	35 kDa		unknown	0	0	0	5	0	0	0	0	0	0	0	0	0
345	TRUE	Empty	malate/lactate/ureidoglycolate dehydr	WP_000253499.1	39 kDa		unknown	0	0	0	5	0	0	0	0	0	0	0	0	0
346	TRUE	Empty	ribonucleotide-diphosphate reductase	WP_000777941.1	36 kDa		unknown	0	0	0	3	0	0	0	0	0	0	0	0	0
347	TRUE	Empty	molecular chaperone HtpG [Escherichia	WP_000678189.1	71 kDa		unknown	0	0	0	0	0	0	0	0	0	2	0	0	2
348	TRUE	Empty	phosphatase [Escherichia coli]	WP_000283657.1	27 kDa		unknown	0	0	3	0	0	0	0	0	0	0	0	0	0
349	TRUE	Empty	MULTISPECIES: cytosol aminopeptidase	WP_000397144.1	55 kDa		unknown	0	0	0	0	0	0	2	0	4	0	0	0	0
350	TRUE	Empty	MULTISPECIES: protease [Enterobacter	WP_000422059.1	39 kDa		unknown	0	0	0	5	0	0	0	0	0	1	0	0	0
351	TRUE	Empty	transcriptional regulator NanR [Escheri	WP_000074798.1	30 kDa		unknown	0	0	0	0	0	0	5	0	0	0	0	0	0
352	TRUE	Empty	MULTISPECIES: NAD(P)H-flavin reducta	WP_000209826.1	26 kDa		unknown	0	0	5	0	0	0	0	0	0	0	0	0	0
353	TRUE	Empty	MULTISPECIES: thiamine ABC transport	WP_000916297.1	25 kDa		unknown	0	0	5	0	0	0	0	0	0	0	0	0	0
354	TRUE	Empty	hypothetical protein [Escherichia coli]	WP_000918421.1	40 kDa		unknown	0	0	0	5	0	0	0	0	0	0	0	0	0
355	TRUE	Empty	RNA-binding transcriptional accessory	WP_000980730.1	85 kDa		unknown	0	0	0	0	0	0	0	0	0	0	5	0	0
356	TRUE	Empty	EscV/YscV/HrcV family type III secretio	WP_001037821.1	75 kDa		unknown	0	0	0	0	0	0	0	0	0	0	4	0	0
357	TRUE	Empty	MULTISPECIES: 16S rRNA pseudouridin	WP_001234850.1	26 kDa		unknown	0	0	0	0	0	0	0	0	5	0	0	0	0
358	TRUE	Empty	SepL/TyeA/HrpJ family type III secretio	WP_001273446.1	40 kDa		unknown	0	0	0	5	0	0	0	0	0	0	0	0	0
359	TRUE	Empty	MULTISPECIES: glutamine--fructose-6-p	WP_000334086.1	67 kDa		unknown	0	0	0	0	1	0	0	0	0	4	0	0	0
360	TRUE	Empty	MULTISPECIES: envelope biogenesis fa	WP_000899583.1	29 kDa		unknown	0	0	0	0	0	0	5	0	0	0	0	0	0
361	TRUE	Empty	MULTISPECIES: molybdenum-depende	WP_001147445.1	28 kDa		unknown	0	0	0	0	0	0	5	0	0	0	0	0	0
362	TRUE	Empty	MULTISPECIES: Lon protease [Proteoba	WP_001295325.1	87 kDa		unknown	0	0	0	0	2	0	0	0	0	0	3	0	0
363	TRUE	Empty	MULTISPECIES: nucleotide exchange fa	WP_001296310.1	22 kDa		unknown	0	0	4	0	0	0	0	0	0	0	0	0	0
364	TRUE	Empty	pyrroline-5-carboxylate reductase [Esc	WP_001339242.1	28 kDa		unknown	0	0	0	0	0	0	5	0	0	0	0	0	0
365	TRUE	Empty	transcriptional regulator SgrR [Escheric	WP_001339533.1	64 kDa		unknown	0	0	0	0	0	0	0	0	0	4	0	0	0
366	TRUE	Empty	MULTISPECIES: elongation factor Ts [Pr	WP_000818114.1	30 kDa		unknown	0	0	4	0	0	0	0	0	1	0	0	0	0
367	TRUE	Empty	hypothetical protein [Escherichia coli]	WP_001103698.1	36 kDa		unknown	0	0	0	0	0	0	2	0	0	0	0	0	0
368	TRUE	Empty	glutamate--cysteine ligase [Escherichia	WP_000611792.1	58 kDa		unknown	0	0	0	0	0	0	0	0	0	4	0	0	0
369	TRUE	Empty	MULTISPECIES: UDP-N-acetylglucosam	WP_000357259.1	45 kDa		unknown	0	0	0	2	0	0	0	0	0	2	0	0	0
370	TRUE	Empty	ATPase AAA [Escherichia coli]	WP_000248600.1	40 kDa		unknown	0	0	1	3	0	0	0	0	0	0	0	0	0
371	TRUE	Empty	MULTISPECIES: adenylosuccinate synth	WP_000527955.1	47 kDa		unknown	0	0	3	2	0	0	0	0	0	0	0	0	0
372	TRUE	Empty	MULTISPECIES: cell division protein Fts	WP_000588474.1	45 kDa		unknown	0	0	0	0	0	0	5	0	0	0	0	0	0
373	TRUE	Empty	membrane protein [Escherichia coli]	WP_000876274.1	11 kDa		unknown	0	5	0	0	0	0	0	0	0	0	0	0	0
374	TRUE	Empty	phosphoribosylglycinamide formyltran	WP_001028627.1	23 kDa		unknown	0	0	4	0	0	0	0	0	0	0	0	0	0
375	TRUE	Empty	MULTISPECIES: beta-ketoacyl-[acyl-car	WP_000044679.1	43 kDa		unknown	0	0	0	0	1	0	0	0	2	2	0	0	0
376	TRUE	Empty	MULTISPECIES: D-aminoacyl-tRNA dea	WP_000560983.1	16 kDa		unknown	0	4	0	0	0	0	0	0	0	0	0	0	0
377	TRUE	Empty	MULTISPECIES: 50S ribosomal protein	WP_000124850.1	13 kDa		unknown	0	3	0	0	0	0	2	0	0	0	0	0	0
378	TRUE	Empty	thiol reductant ABC exporter subunit C	WP_001043562.1	65 kDa		unknown	0	0	0	0	0	0	0	0	0	4	0	0	0
379	TRUE	Empty	NAD(+) synthetase [Escherichia coli]	WP_000175022.1	31 kDa		unknown	0	0	0	4	0	0	0	0	0	0	0	0	0
380	TRUE	Empty	MULTISPECIES: Low-affinity inorganic p	WP_000902780.1	53 kDa		unknown	0	0	0	0	0	0	0	0	0	0	4	0	0
381	TRUE	Empty	MULTISPECIES: UDP-N-acetyl-D-manno	WP_001064027.1	28 kDa		unknown	0	0	4	0	0	0	0	0	0	0	0	0	0
382	TRUE	Empty	DNA-binding response regulator [Esche	WP_000186067.1	25 kDa		unknown	0	0	4	0	0	0	0	0	0	0	0	0	0
383	TRUE	Empty	MULTISPECIES: endonuclease V [Prote	WP_000362388.1	25 kDa		unknown	0	0	3	0	0	0	0	0	0	0	0	0	0
384	TRUE	Empty	hypothetical protein [Escherichia coli]	WP_001121571.1	25 kDa		unknown	0	0	3	0	0	0	0	0	0	0	0	0	0
385	TRUE	Empty	cell division protein [Escherichia coli]	WP_000279544.1	28 kDa		unknown	0	0	4	0	0	0	0	0	0	0	0	0	0
386	TRUE	Empty	MULTISPECIES: DNA topoisomerase IV	WP_001281888.1	84 kDa		unknown	0	0	0	0	0	0	0	0	0	0	4	0	0
387	TRUE	Empty	glutamate synthase [Escherichia coli]	WP_001409556.1	163 kDa		unknown	0	0	0	0	1	0	0	0	0	0	2	0	0
388	TRUE	Empty	MULTISPECIES: protein YebE [Escherich	WP_000024725.1	24 kDa		unknown	0	0	4	0	0	0	0	0	0	0	0	0	0
389	TRUE	Empty	MULTISPECIES: ferredoxin-type protein	WP_000091291.1	25 kDa		unknown	0	0	0	0	0	0	4	0	0	0	0	0	0
390	TRUE	Empty	AraC family transcriptional regulator [E	WP_000274618.1	32 kDa		unknown	0	0	0	0	0	0	0	0	4	0	0	0	0
391	TRUE	Empty	MULTISPECIES: aminoglycoside O-phos	WP_000480968.1	31 kDa		unknown	0	0	0	0	0	0	4	0	0	0	0	0	0
392	TRUE	Empty	MULTISPECIES: phosphate ABC transpo	WP_000867149.1	37 kDa		unknown	0	0	0	4	0	0	0	0	0	0	0	0	0
393	TRUE	Empty	MULTISPECIES: magnesium transporte	WP_000947159.1	37 kDa		unknown	0	0	0	4	0	0	0	0	0	0	0	0	0
394	TRUE	Empty	tail assembly protein [Escherichia coli]	WP_000978925.1	17 kDa		unknown	0	4	0	0	0	0	0	0	0	0	0	0	0
395	TRUE	Empty	MULTISPECIES: ribonuclease 3 [Proteol	WP_001068343.1	26 kDa		unknown	0	0	4	0	0	0	0	0	0	0	0	0	0
396	TRUE	Empty	MULTISPECIES: aminoalkylphosphonic	WP_001110514.1	17 kDa		unknown	0	4	0	0	0	0	0	0	0	0	0	0	0
397	TRUE	Empty	DNA-binding transcriptional regulator /	WP_001298645.1	32 kDa		unknown	0	0	0	0	0	0	0	0	4	0	0	0	0
398	TRUE	Empty	DNA-processing protein DprA [Escheric	WP_001324531.1	36 kDa		unknown	0	0	0	3	0	0	0	0	0	0	0	0	0
399	TRUE	Empty	MULTISPECIES: transcription terminati	WP_001287521.1	21 kDa		unknown	0	3	0	0	0	0	0	0	0	0	0	0	0
400	TRUE	Empty	MULTISPECIES: nucleoside permease N	WP_000376337.1	43 kDa		unknown	0	0	0	0	0	0	0	0	0	0	4	0	0
401	TRUE	Empty	deferrochelatase/peroxidase YfeX [Esc	WP_001339829.1	33 kDa		unknown	0	0	0	0	0	0	0	0	2	0	0	0	0

402	TRUE	Empty	accessory colonization factor AcfD [Esc	WP_001340008.1	168 kDa		unknown	0	0	0	0	0	0	0	0	0	0	3	0	0
403	TRUE	Empty	MULTISPECIES: phage shock protein Ps	WP_000511017.1	26 kDa		unknown	0	0	2	0	0	0	0	0	0	0	0	0	0
404	TRUE	Empty	MULTISPECIES: glycerol kinase [Entero	WP_000136804.1	56 kDa		unknown	0	0	0	0	0	0	0	0	0	3	0	0	0
405	TRUE	Empty	2-succinyl-5-enolpyruvyl-6-hydroxy-3-c	WP_001339807.1	61 kDa		unknown	0	0	0	0	0	0	0	0	0	3	0	0	0
406	TRUE	Empty	transcriptional regulator [Escherichia c	WP_000018007.1	23 kDa		unknown	0	0	0	0	0	0	1	0	2	0	0	0	0
407	TRUE	Empty	MULTISPECIES: DNA-binding protein [E	WP_000115381.1	15 kDa		unknown	0	2	0	0	0	0	0	0	0	0	0	0	0
408	TRUE	Empty	MULTISPECIES: peptide transport syste	WP_000573407.1	31 kDa		unknown	0	0	0	0	0	0	0	0	3	0	0	0	0
409	TRUE	Empty	MULTISPECIES: UTP--glucose-1-phosph	WP_000718995.1	33 kDa		unknown	0	0	0	3	0	0	0	0	0	0	0	0	0
410	TRUE	Empty	MULTISPECIES: histidine ABC transport	WP_000737621.1	28 kDa		unknown	0	0	0	0	0	0	3	0	0	0	0	0	0
411	TRUE	Empty	MULTISPECIES: molybdopterin-synthas	WP_000829253.1	27 kDa		unknown	0	0	3	0	0	0	0	0	0	0	0	0	0
412	TRUE	Empty	MULTISPECIES: peptidylprolyl isomeras	WP_000861331.1	21 kDa		unknown	0	0	3	0	0	0	0	0	0	0	0	0	0
413	TRUE	Empty	MULTISPECIES: endonuclease [Enterob	WP_000873880.1	31 kDa		unknown	0	0	0	0	0	0	3	0	0	0	0	0	0
414	TRUE	Empty	MULTISPECIES: molecular chaperone G	WP_001026276.1	10 kDa		unknown	0	2	0	0	0	0	0	0	0	0	0	0	0
415	TRUE	Empty	AIDA autotransporter [Escherichia coli]	WP_001082402.1	111 kDa		unknown	0	0	0	0	0	0	0	0	0	0	3	0	0
416	TRUE	Empty	hypothetical protein [Escherichia coli]	WP_001102130.1	26 kDa		unknown	0	0	3	0	0	0	0	0	0	0	0	0	0
417	TRUE	Empty	LPS 1,3-galactosyltransferase [Escheric	WP_001188026.1	39 kDa		unknown	0	0	0	3	0	0	0	0	0	0	0	0	0
418	TRUE	Empty	MULTISPECIES: formate dehydrogenas	WP_012579028.1	113 kDa		unknown	0	0	0	0	0	0	0	0	0	0	0	0	3
419	TRUE	Empty	hypothetical protein [Escherichia coli]	WP_000151816.1	23 kDa		unknown	0	2	0	0	0	0	0	0	0	0	0	0	0
420	TRUE	Empty	toxin B [Escherichia coli]	WP_012578873.1	298 kDa		unknown	0	0	0	0	2	0	0	0	0	0	0	0	0
421	TRUE	Empty	MULTISPECIES: uracil-DNA glycosylase	WP_001262723.1	26 kDa		unknown	0	0	3	0	0	0	0	0	0	0	0	0	0
422	TRUE	Empty	MULTISPECIES: ATP phosphoribosyltra	WP_000131782.1	33 kDa		unknown	0	0	0	0	0	0	2	0	0	0	0	0	0
423	TRUE	Empty	MULTISPECIES: two-component systen	WP_000190574.1	39 kDa		unknown	0	0	0	3	0	0	0	0	0	0	0	0	0
424	TRUE	Empty	MULTISPECIES: 2,3-bisphosphoglycerat	WP_001295305.1	29 kDa		unknown	0	0	3	0	0	0	0	0	0	0	0	0	0
425	TRUE	Empty	MULTISPECIES: phosphoglycerate kinas	WP_000111269.1	41 kDa		unknown	0	0	0	2	0	0	1	0	0	0	0	0	0
426	TRUE	Empty	MULTISPECIES: enterotoxin [Escherichi	WP_001121619.1	62 kDa		unknown	0	0	3	0	0	0	0	0	0	0	0	0	0
427	TRUE	Empty	MULTISPECIES: antiporter [Enterobacte	WP_000246011.1	55 kDa		unknown	0	0	0	0	0	0	0	0	0	0	3	0	0
428	TRUE	Empty	adenosine deaminase [Escherichia coli	WP_000567515.1	36 kDa		unknown	0	0	0	2	0	0	0	0	0	0	0	0	0
429	TRUE	Empty	MULTISPECIES: phospholipid-binding li	WP_000776774.1	28 kDa		unknown	0	0	0	0	0	0	2	0	0	0	0	0	0
430	TRUE	Empty	MULTISPECIES: DNA recombination/re	WP_000963143.1	38 kDa		unknown	0	0	0	0	0	0	0	0	2	0	0	0	0
431	TRUE	Empty	pimeloyl-[acyl-carrier protein] methyl e	WP_001060053.1	29 kDa		unknown	0	0	2	0	0	0	0	0	0	0	0	0	0
432	TRUE	Empty	5'/3'-nucleotidase SurE [Escherichia co	WP_001374596.1	27 kDa		unknown	0	0	0	0	0	0	0	0	2	0	0	0	0
433	TRUE	Empty	MULTISPECIES: NAD(P)-dependent oxic	WP_000754737.1	30 kDa		unknown	0	0	0	0	0	0	0	0	2	0	0	0	0
434	TRUE	Empty	MULTISPECIES: chemotaxis protein [En	WP_000203541.1	34 kDa		unknown	0	0	0	0	0	0	2	0	0	0	0	0	0
435	TRUE	Empty	MULTISPECIES: phosphoenolpyruvate c	WP_001265681.1	60 kDa		unknown	0	0	0	0	0	0	0	0	0	2	0	0	0
436	TRUE	Empty	MULTISPECIES: TyrR family transcriptio	WP_001296041.1	58 kDa		unknown	0	0	0	0	0	0	0	0	0	2	0	0	0
437	TRUE	Empty	MULTISPECIES: YraN family protein [En	WP_000246829.1	15 kDa		unknown	0	2	0	0	0	0	0	0	0	0	0	0	0
438	TRUE	Empty	MULTISPECIES: dnaJ-like protein DjlA [I	WP_001200560.1	31 kDa		unknown	0	0	0	0	0	0	2	0	0	0	0	0	0
439	TRUE	Empty	cell division protein CpoB [Escherichia (	WP_000097566.1	28 kDa		unknown	0	0	0	0	0	0	2	0	0	0	0	0	0

END OF FILE

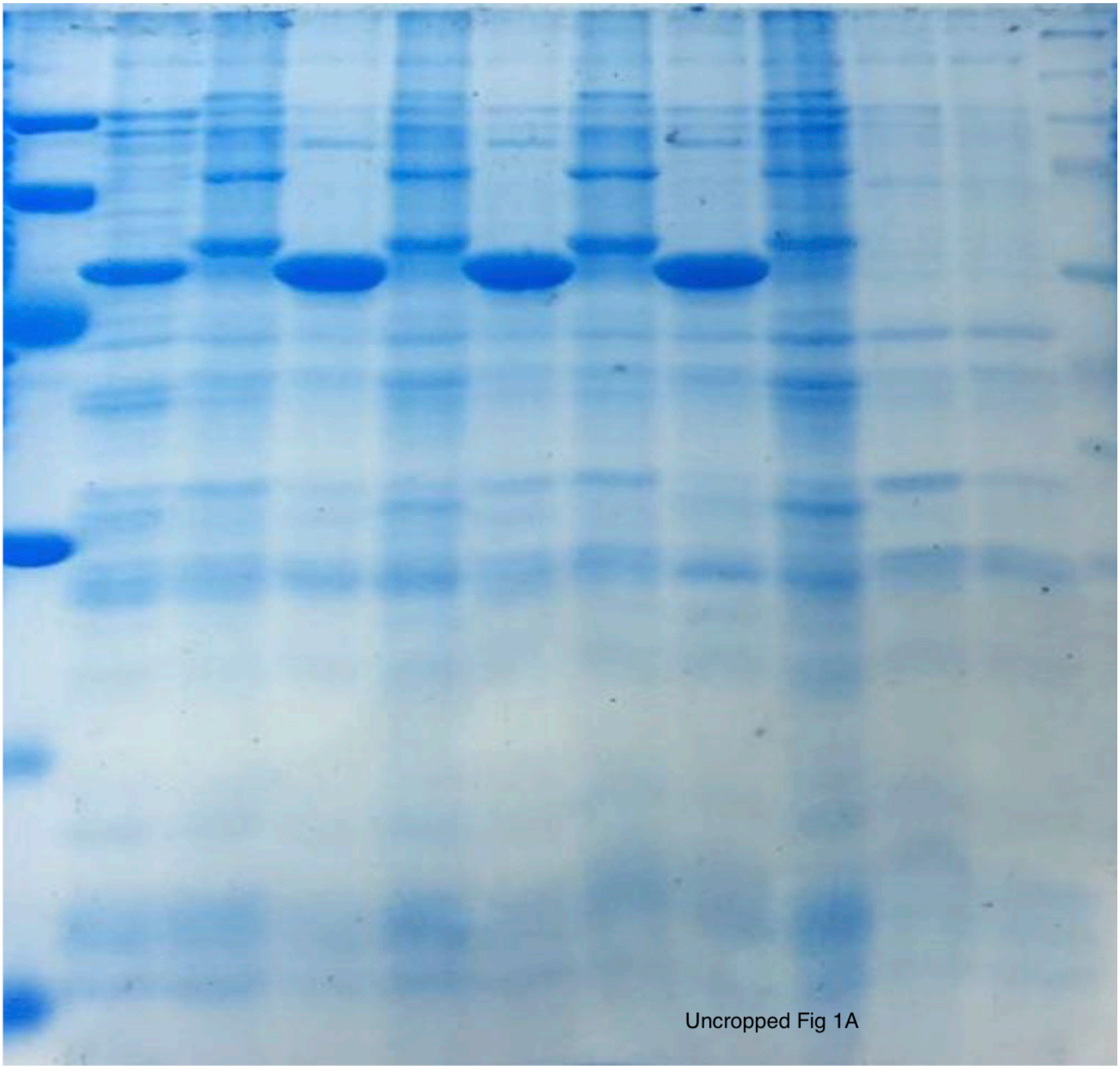
Uncropped Fig 1A

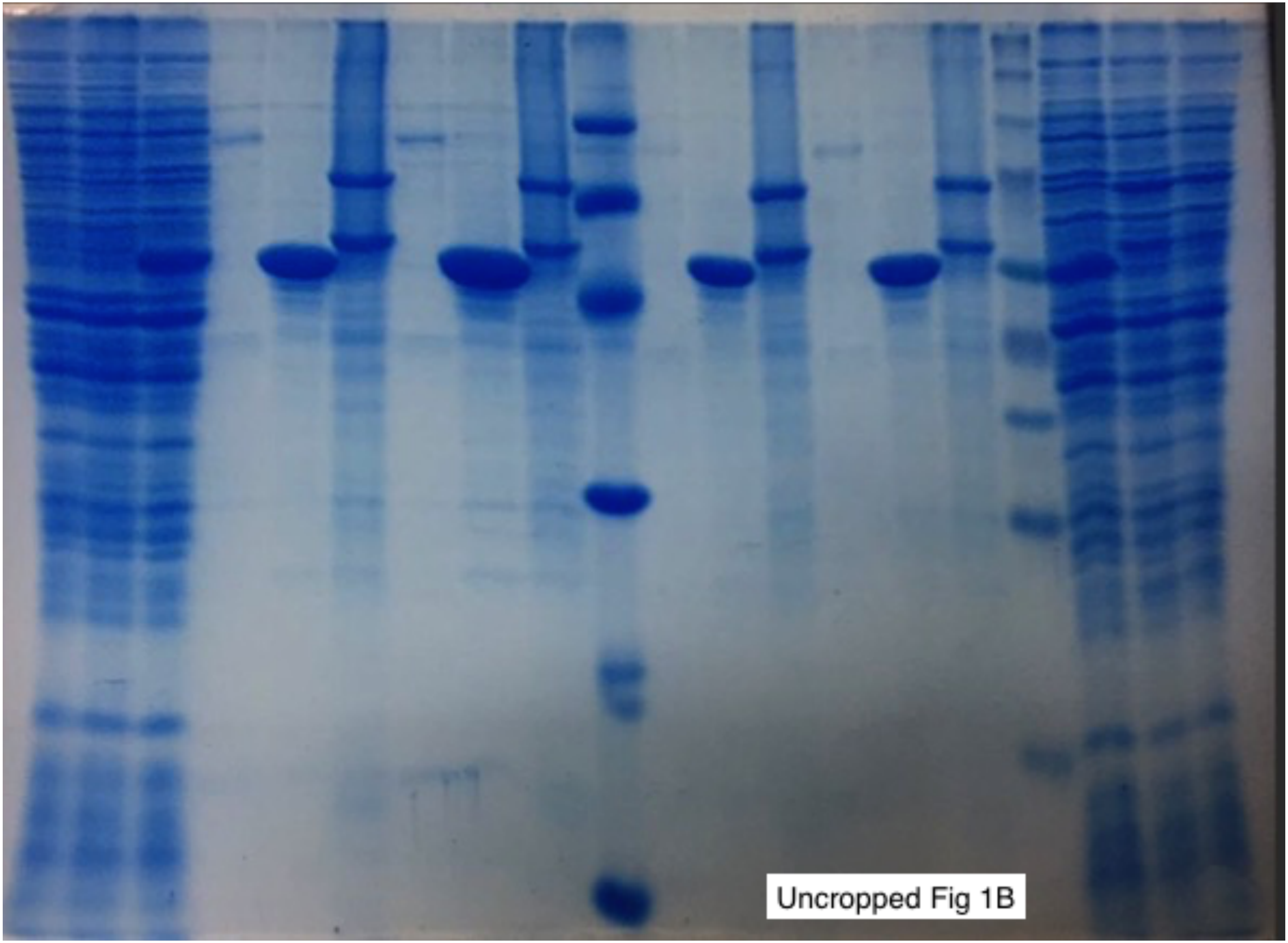
Uncropped Fig 1B

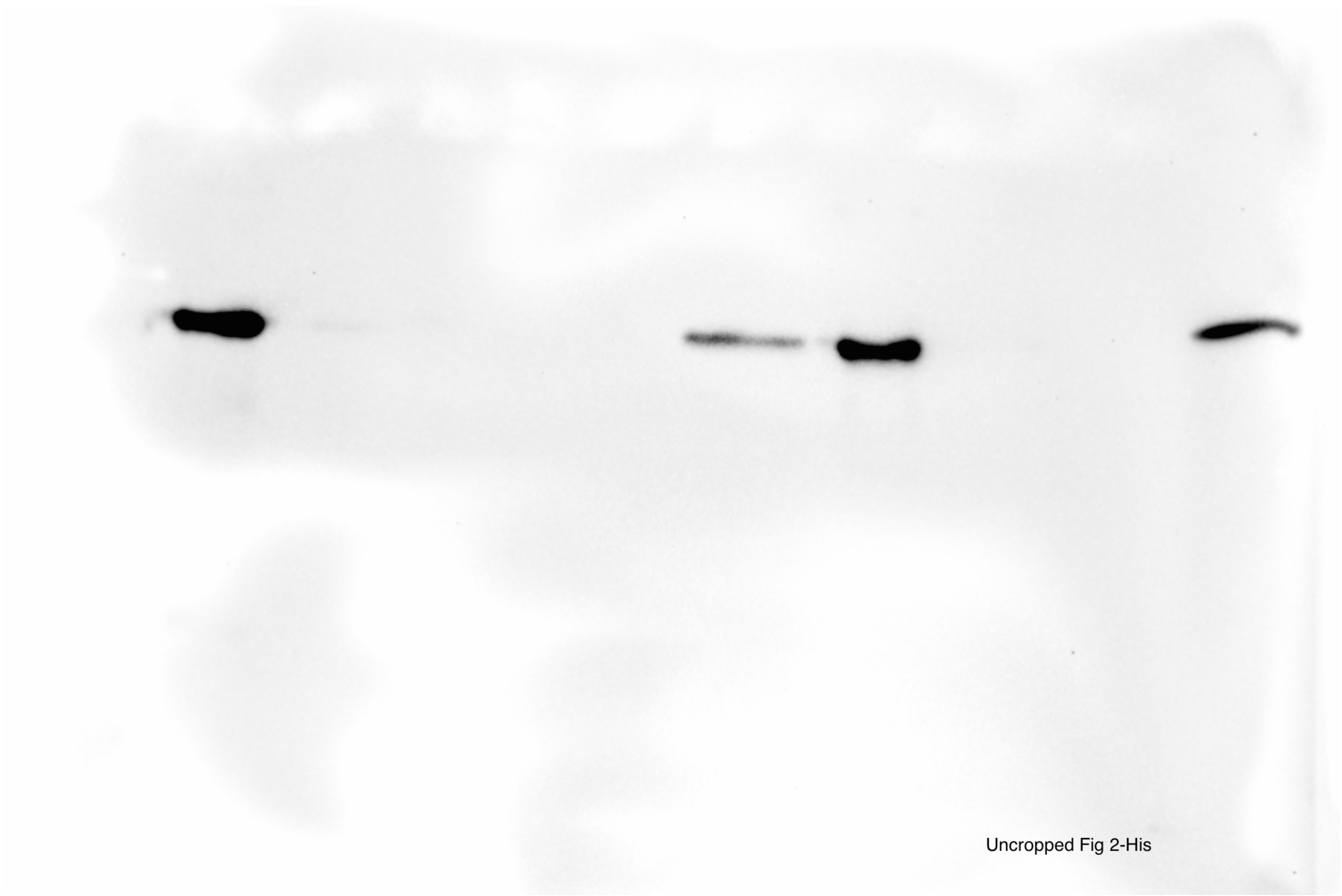

Uncropped Fig 2-His

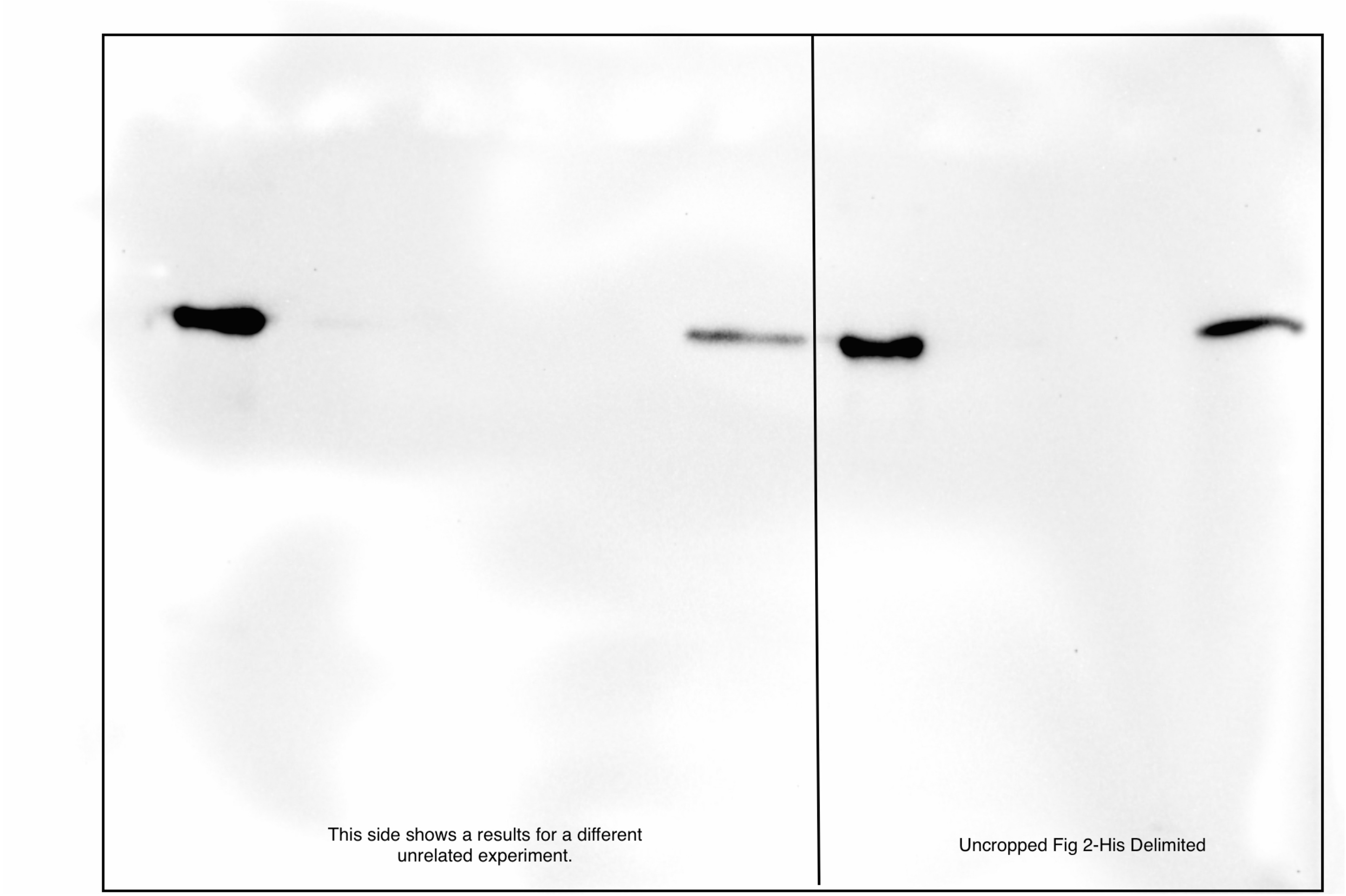
This side shows a results for a different
unrelated experiment.
Uncropped Fig 2-His Delimited

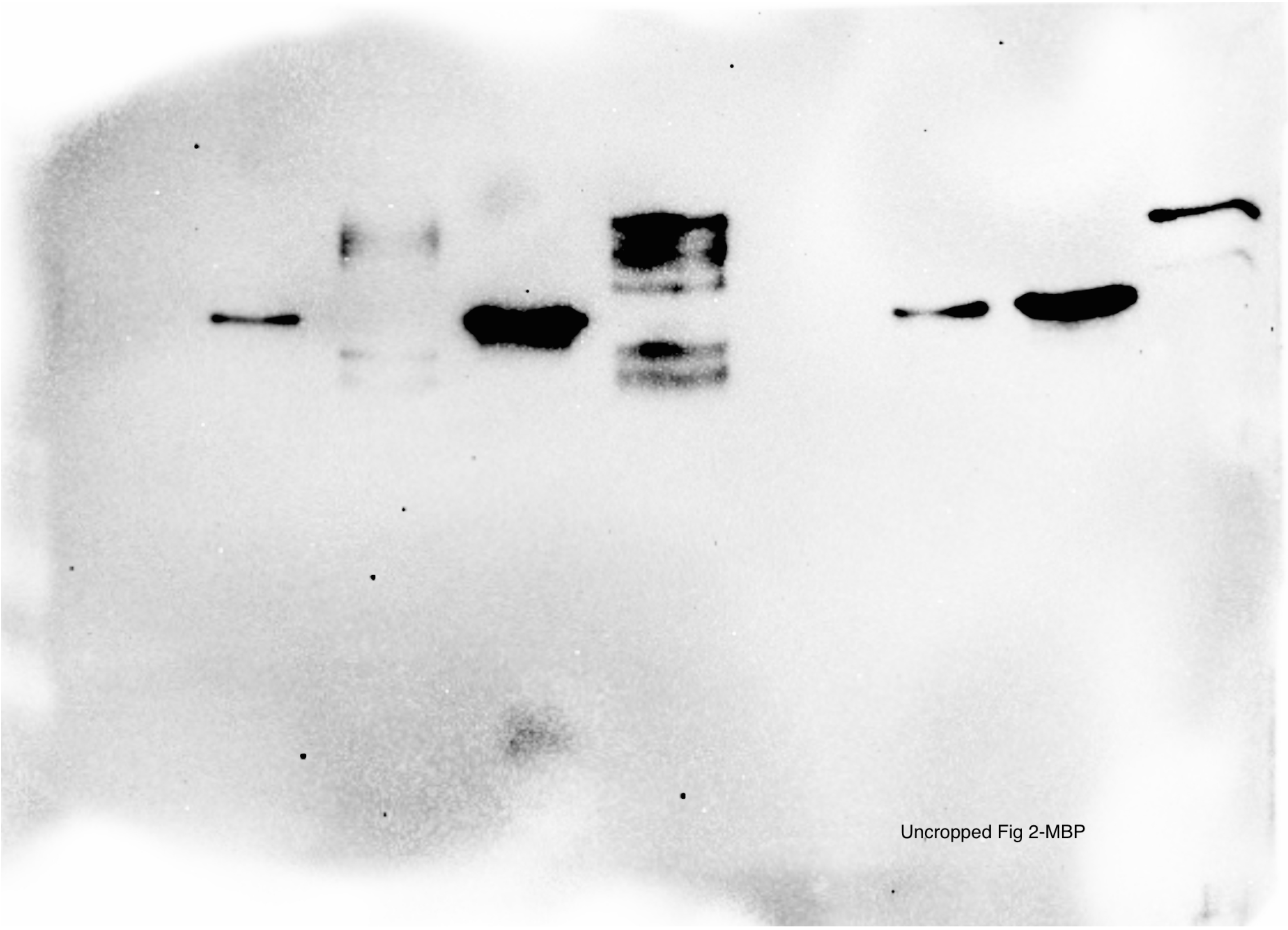

Uncropped Fig 2-MBP

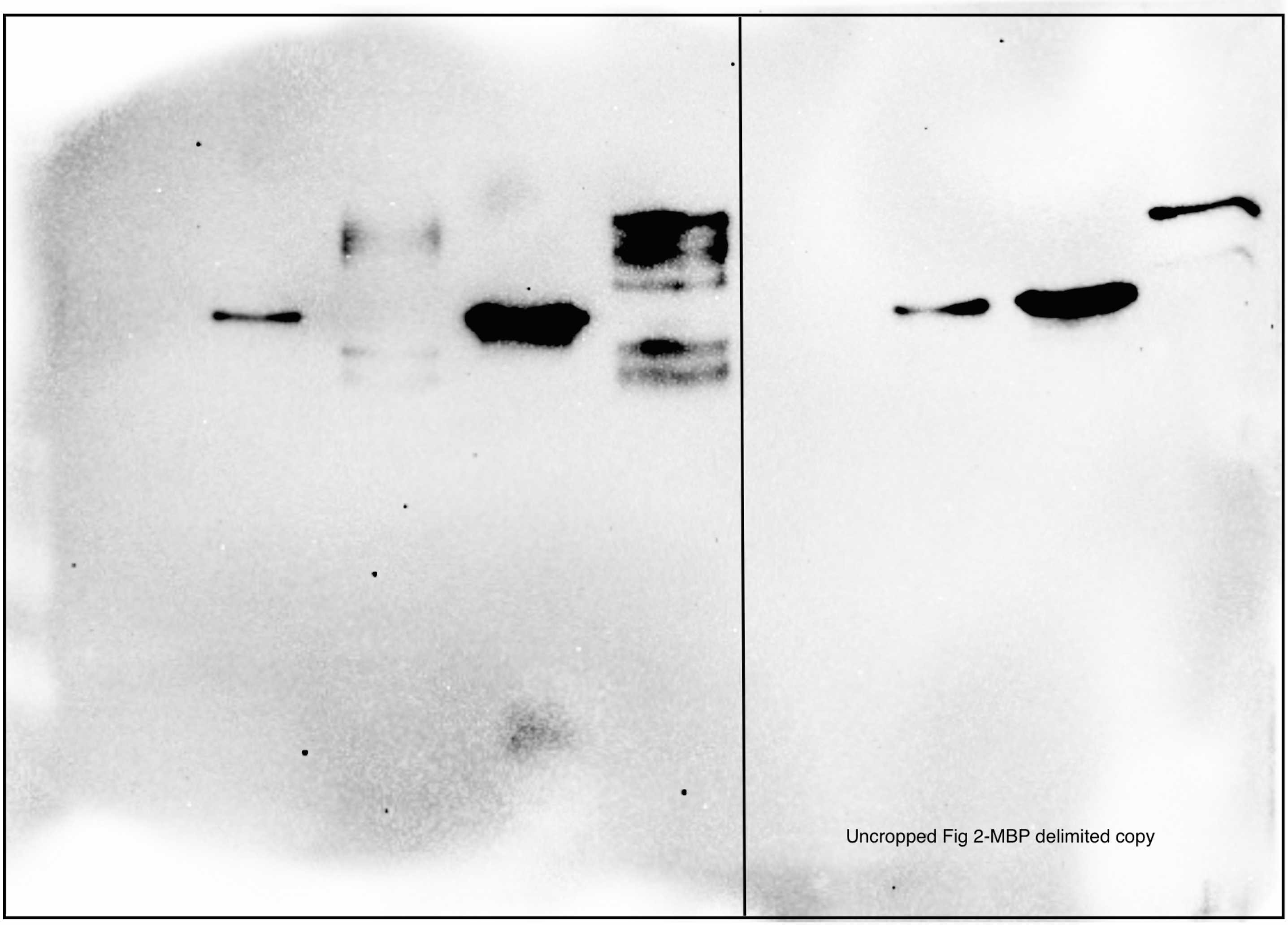
Uncropped Fig 2-MBP delimited copy

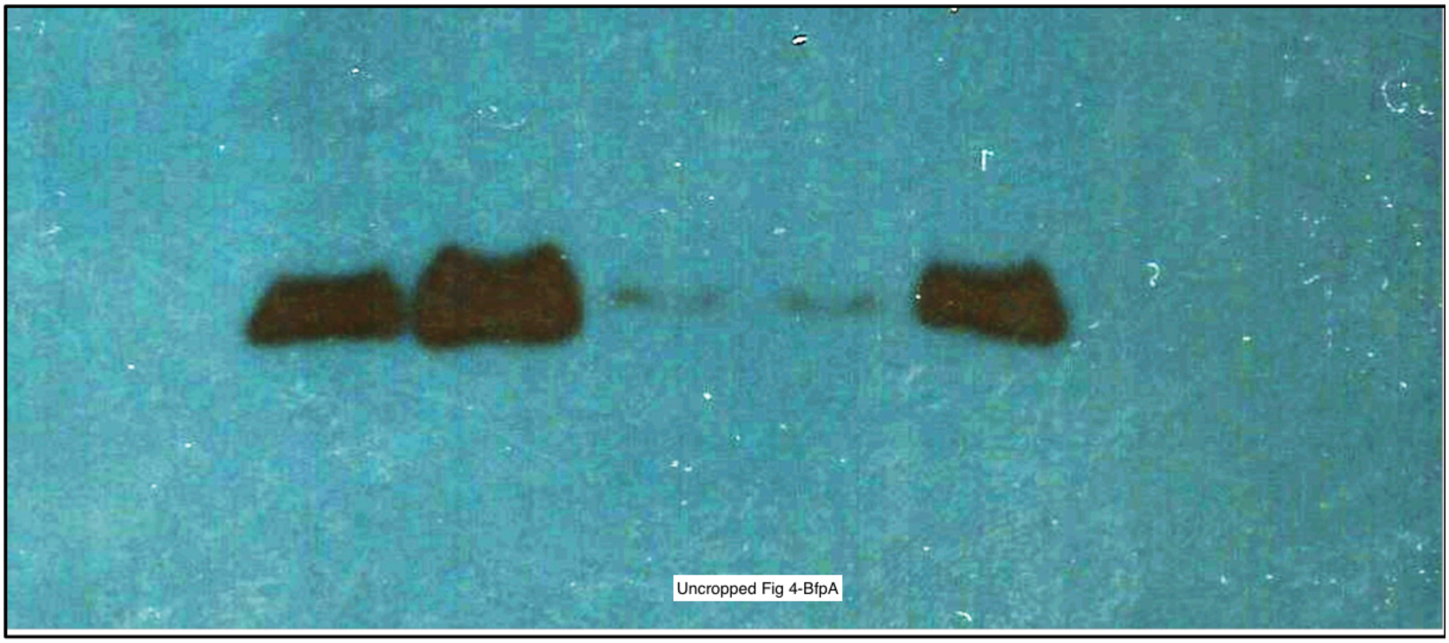
Uncropped Fig 4-BfpA

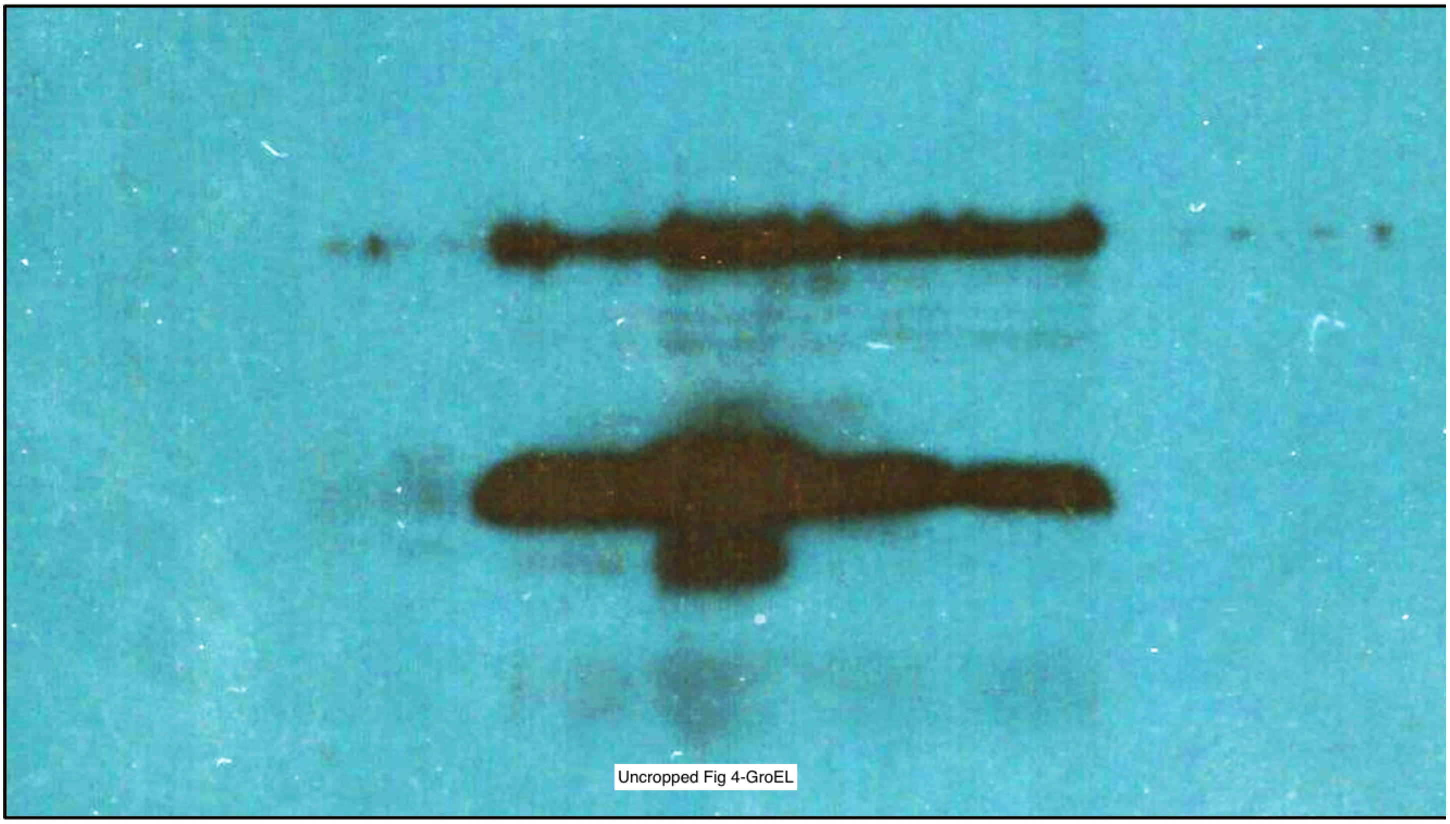
Uncropped Fig 4-GroEL

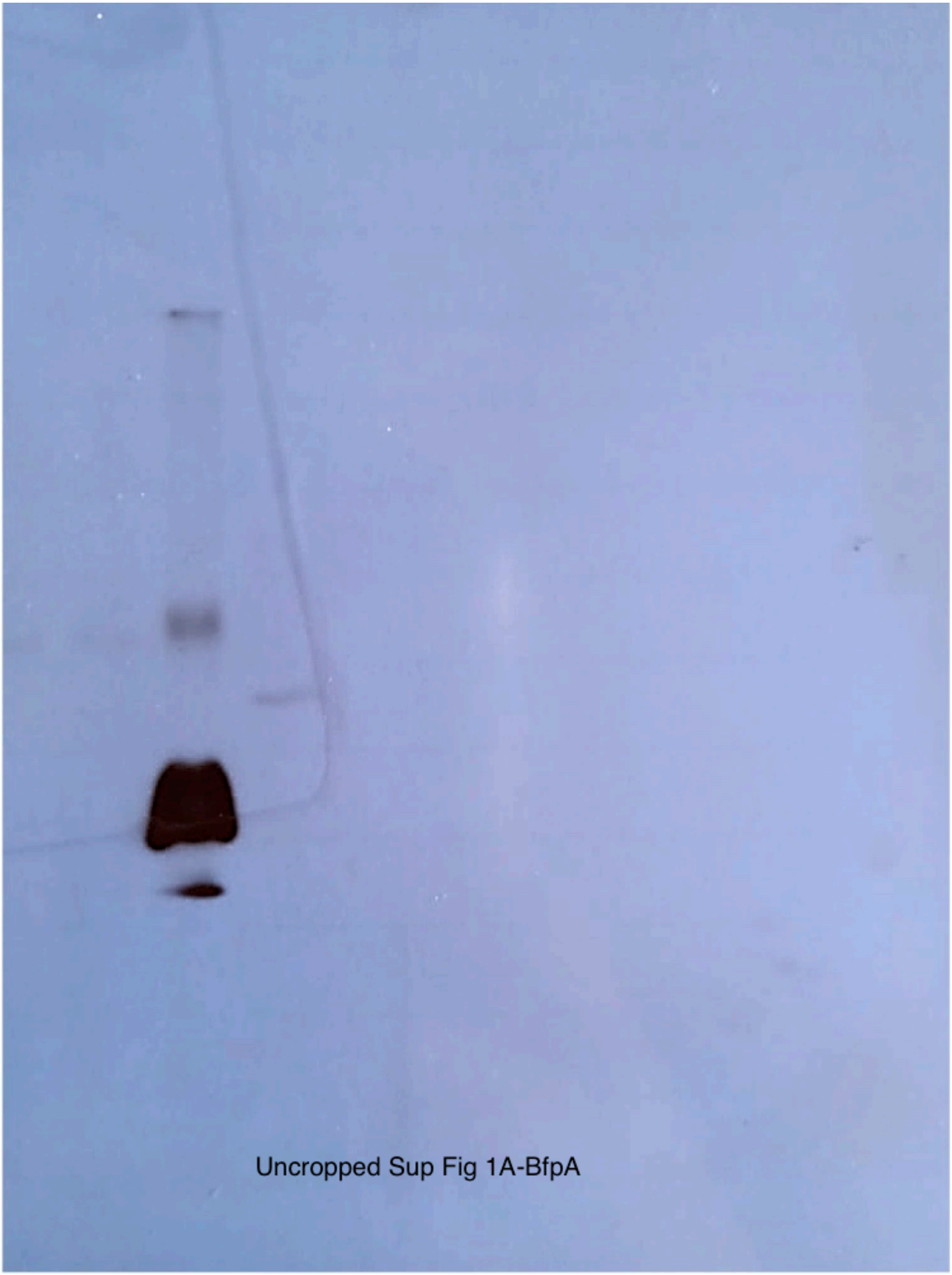

Uncropped Sup Fig 1A-BfpA

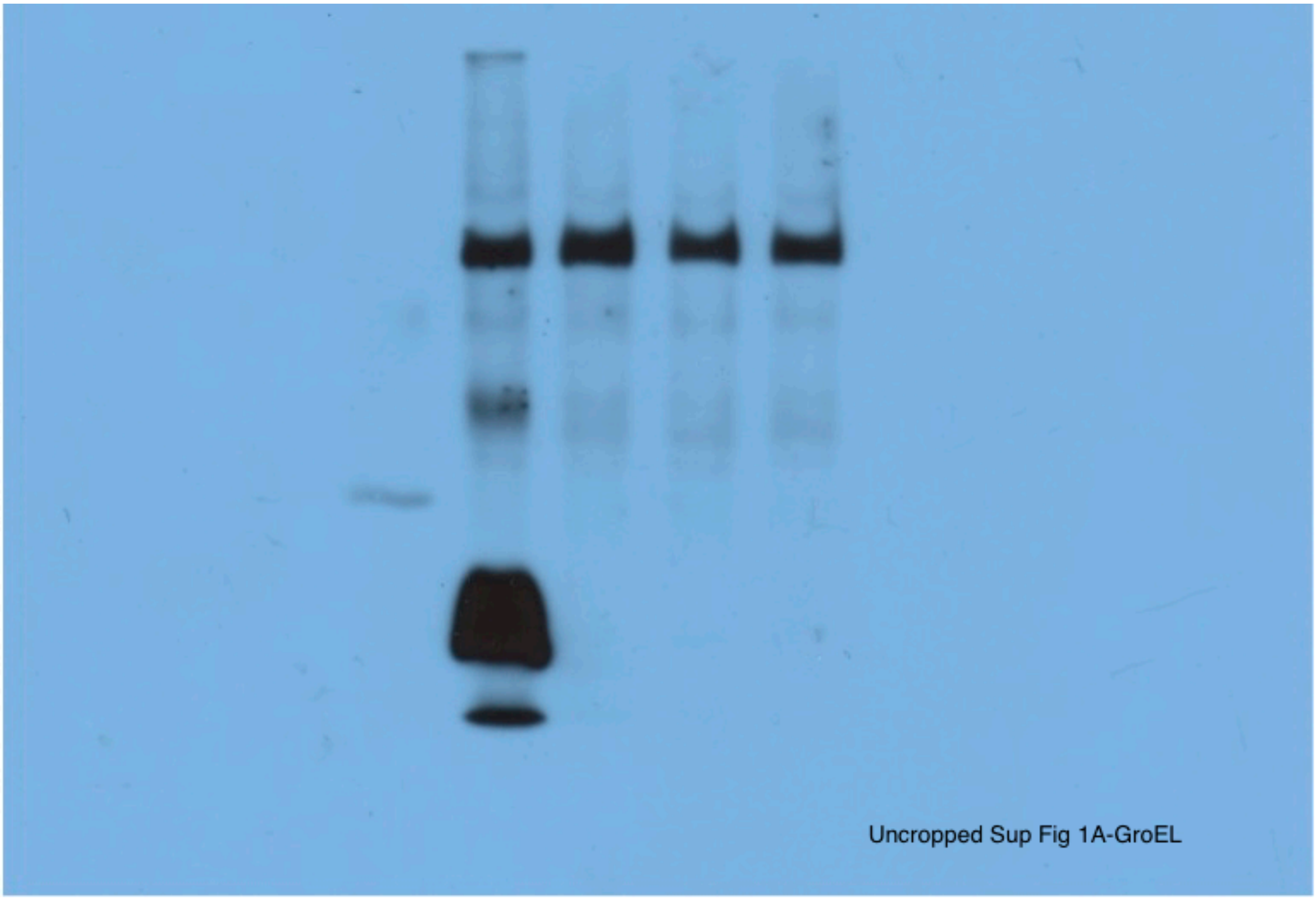
Uncropped Sup Fig 1A-GroEL

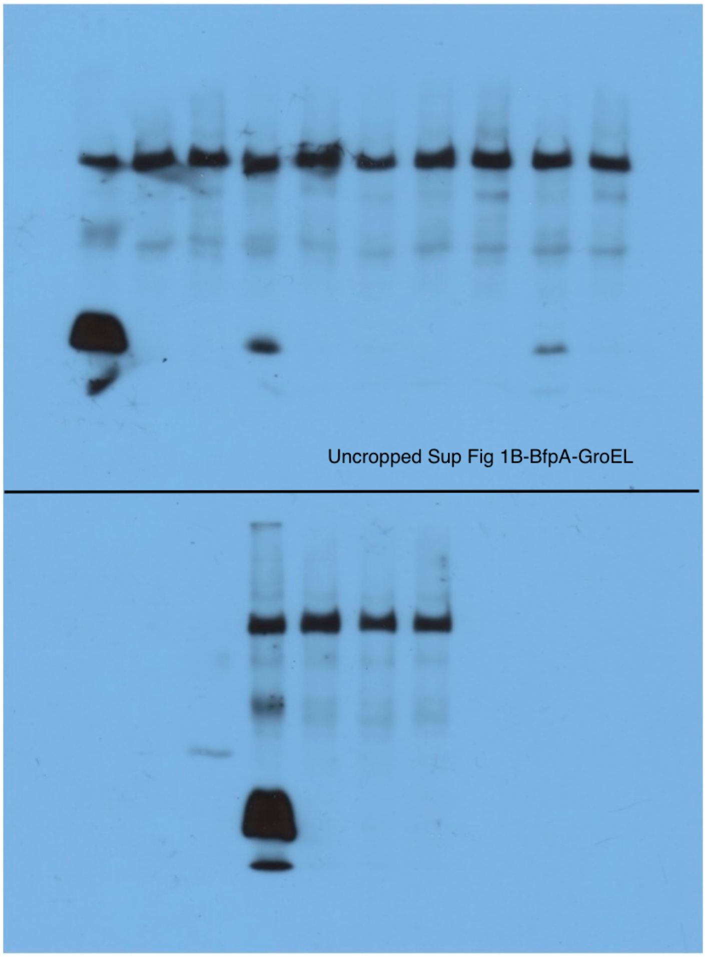

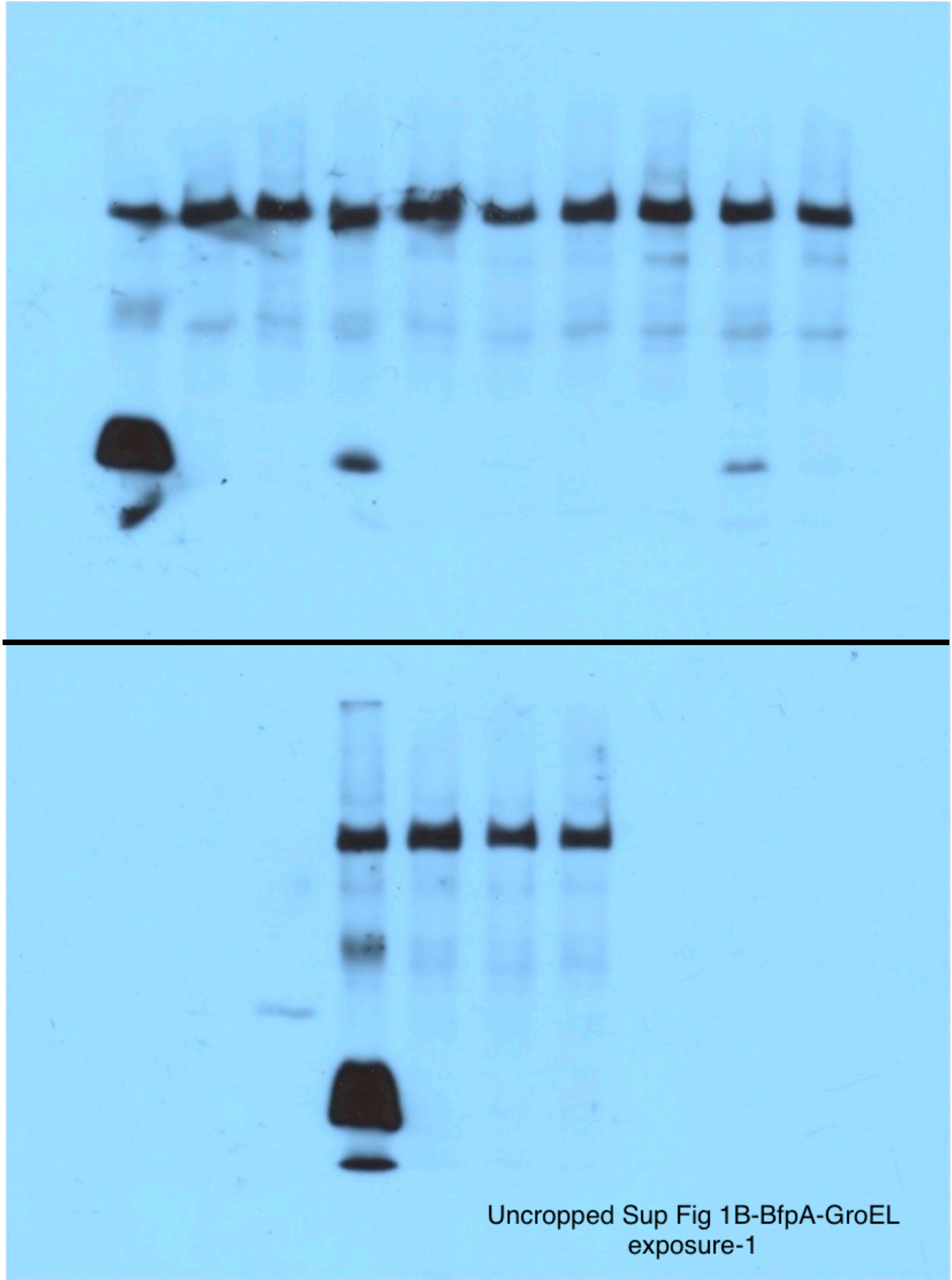

Uncropped Sup Fig 1B-BfpA-GroEL
exposure-1

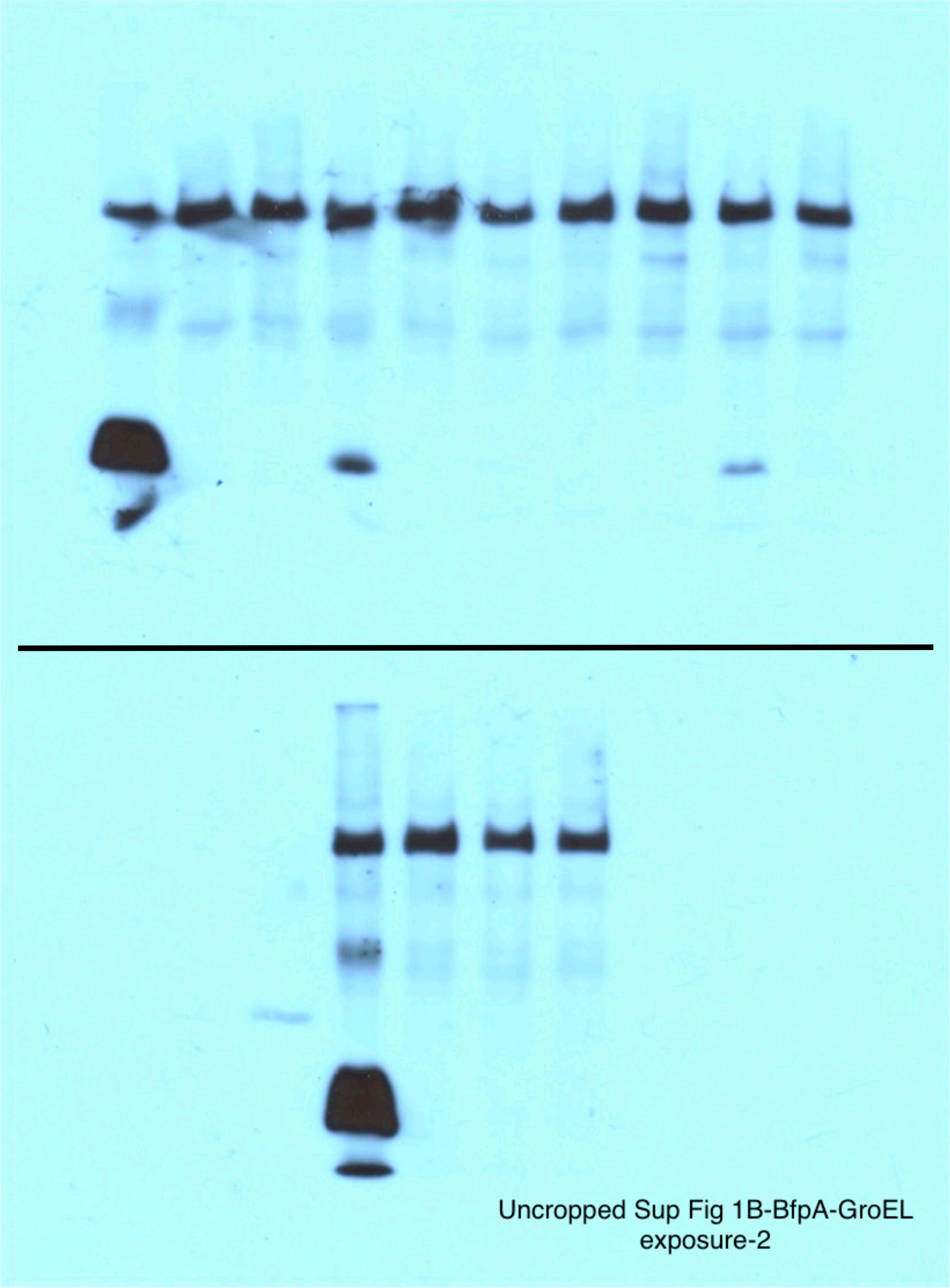

Uncropped Sup Fig 1B-BfpA-GroEL
exposure-2

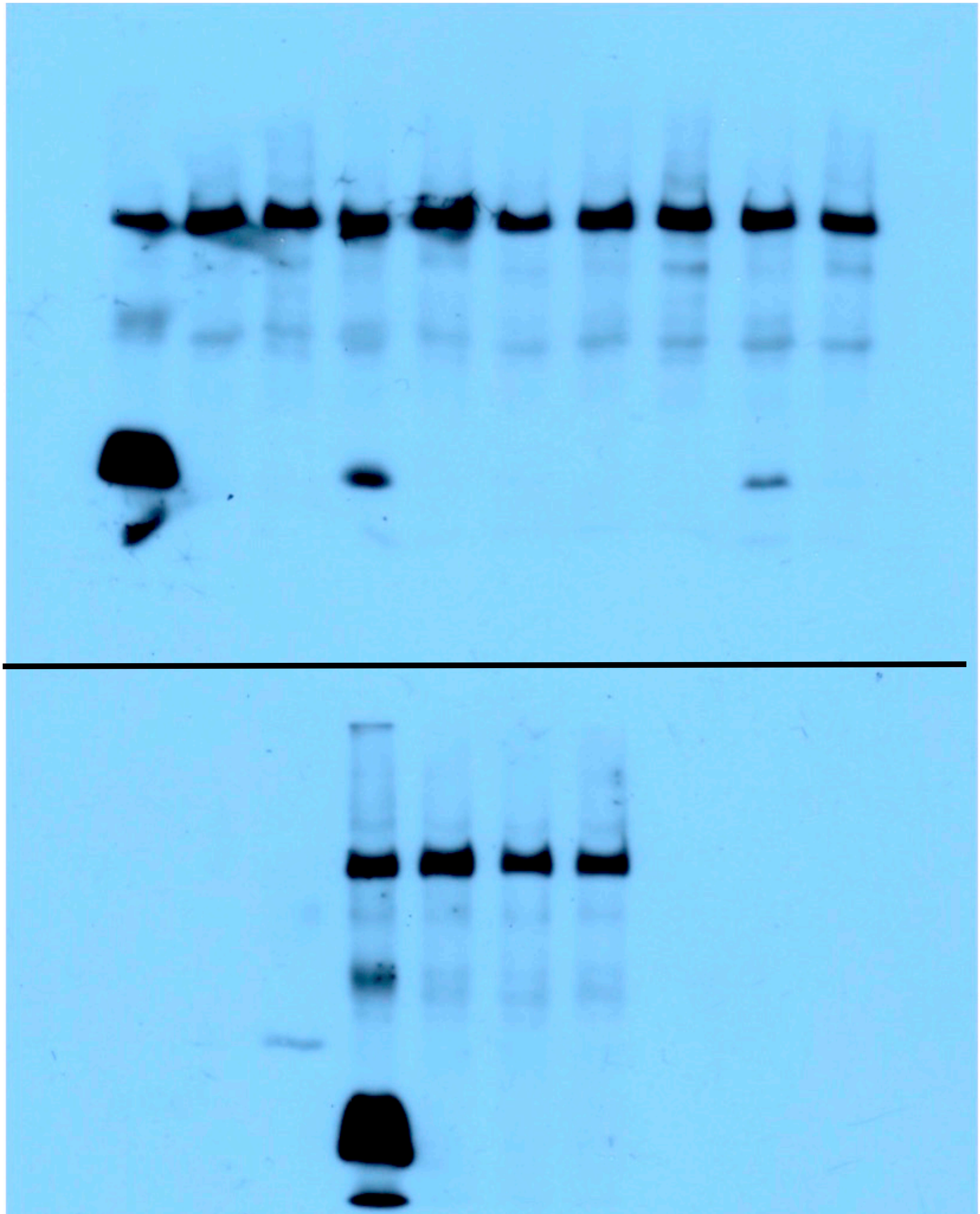

Uncropped Sup Fig 1B-BfpA-GroEL
exposure-3

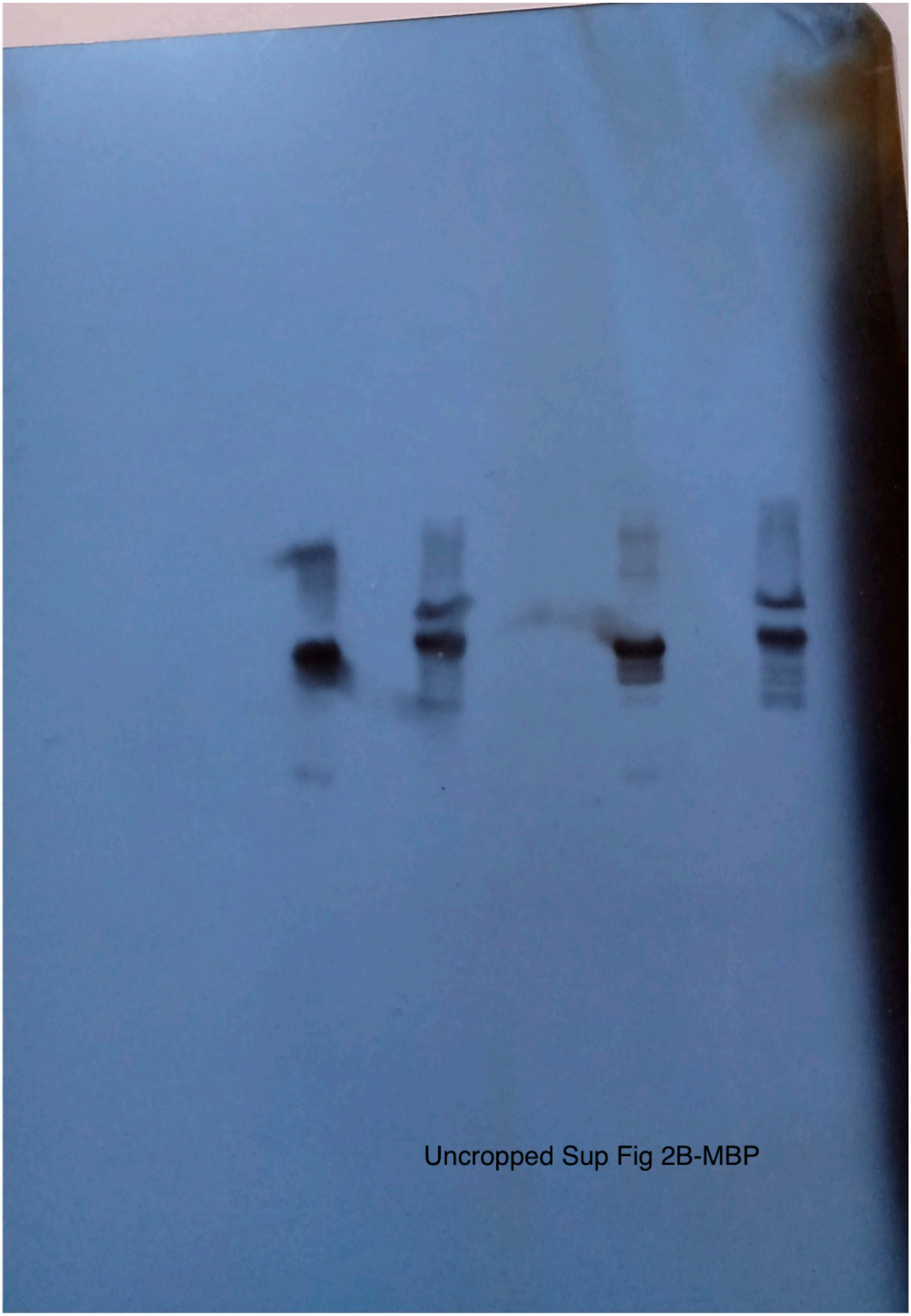

Uncropped Sup Fig 2B-MBP

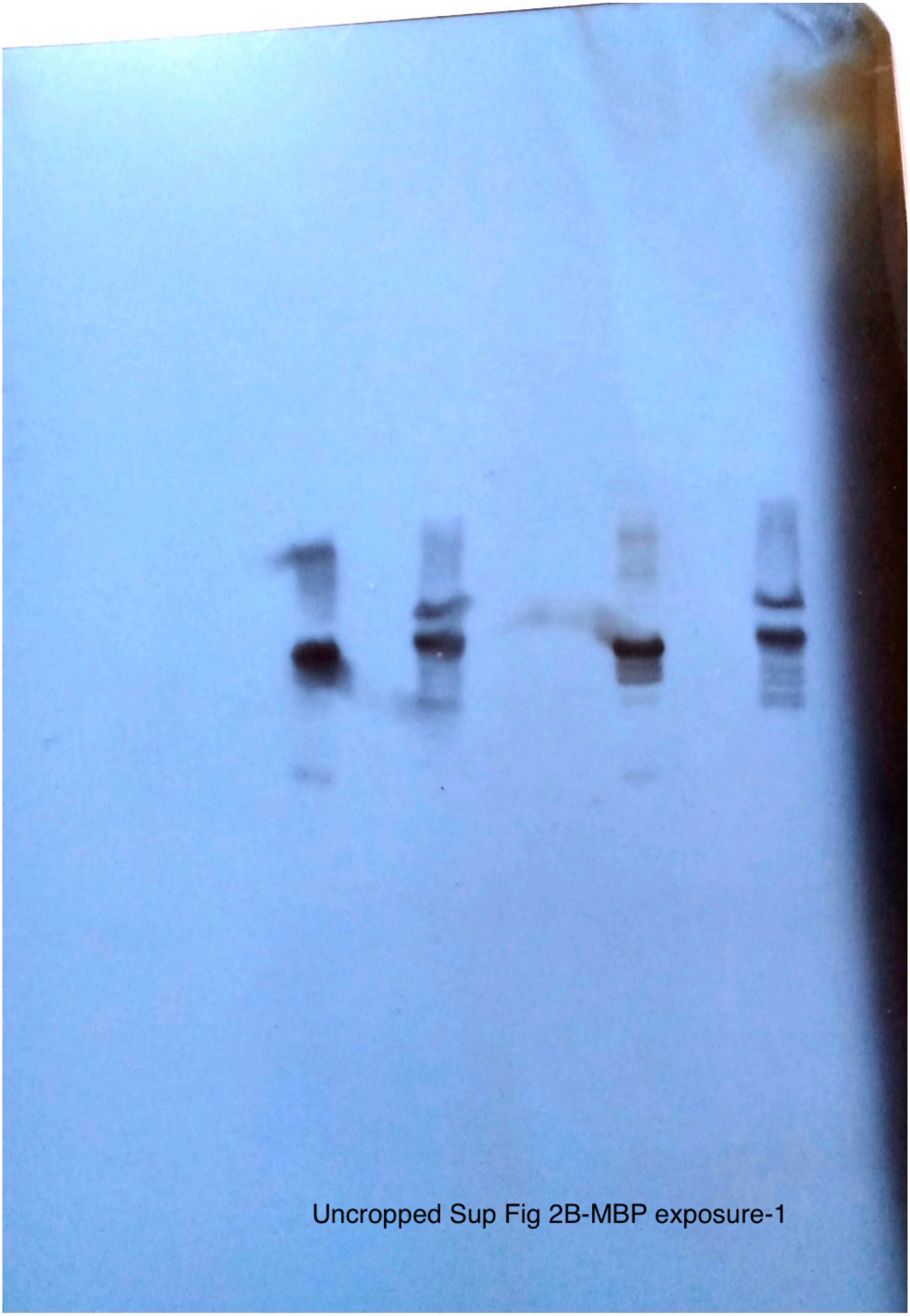

Uncropped Sup Fig 2B-MBP exposure-1

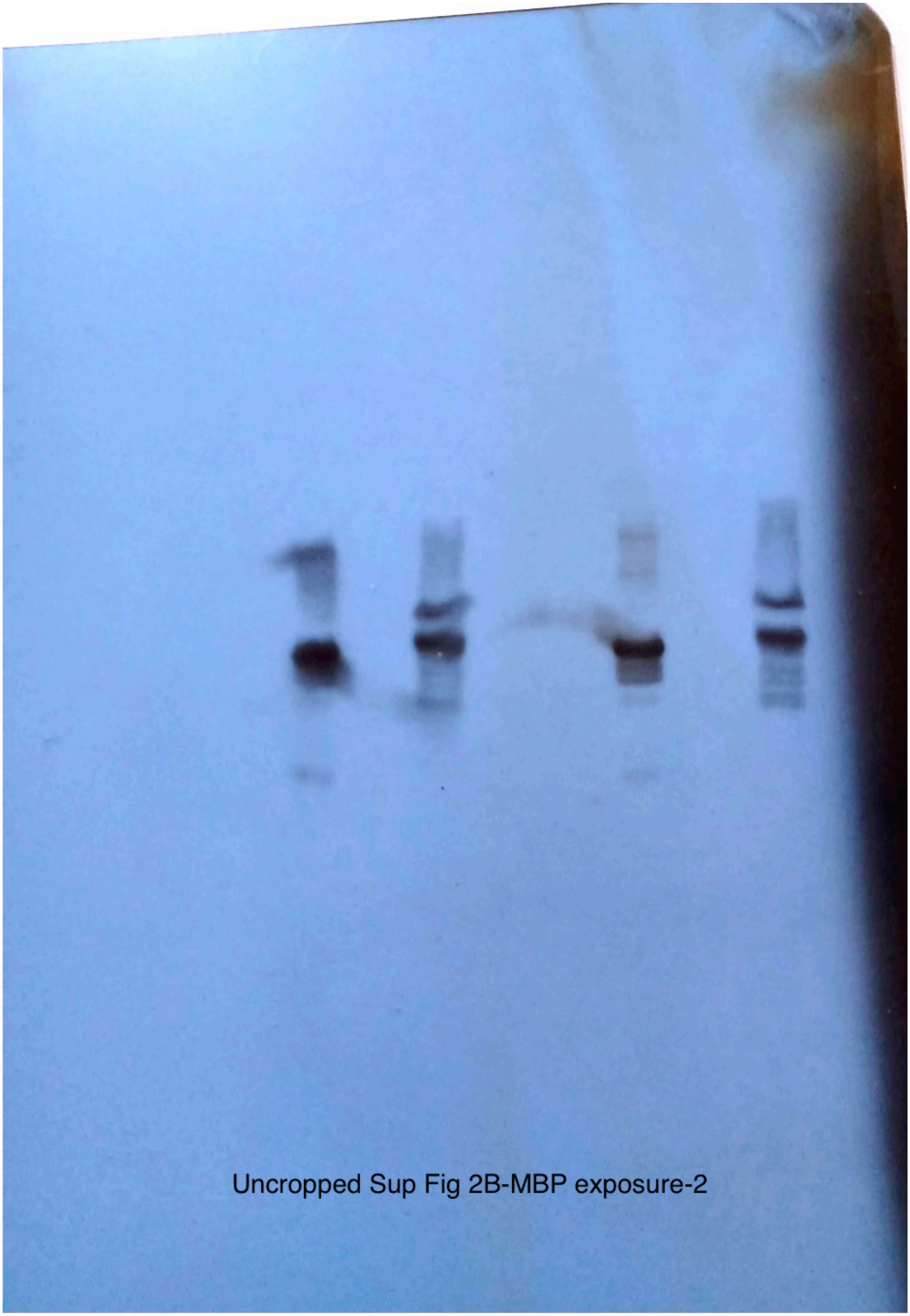

Uncropped Sup Fig 2B-MBP exposure-2

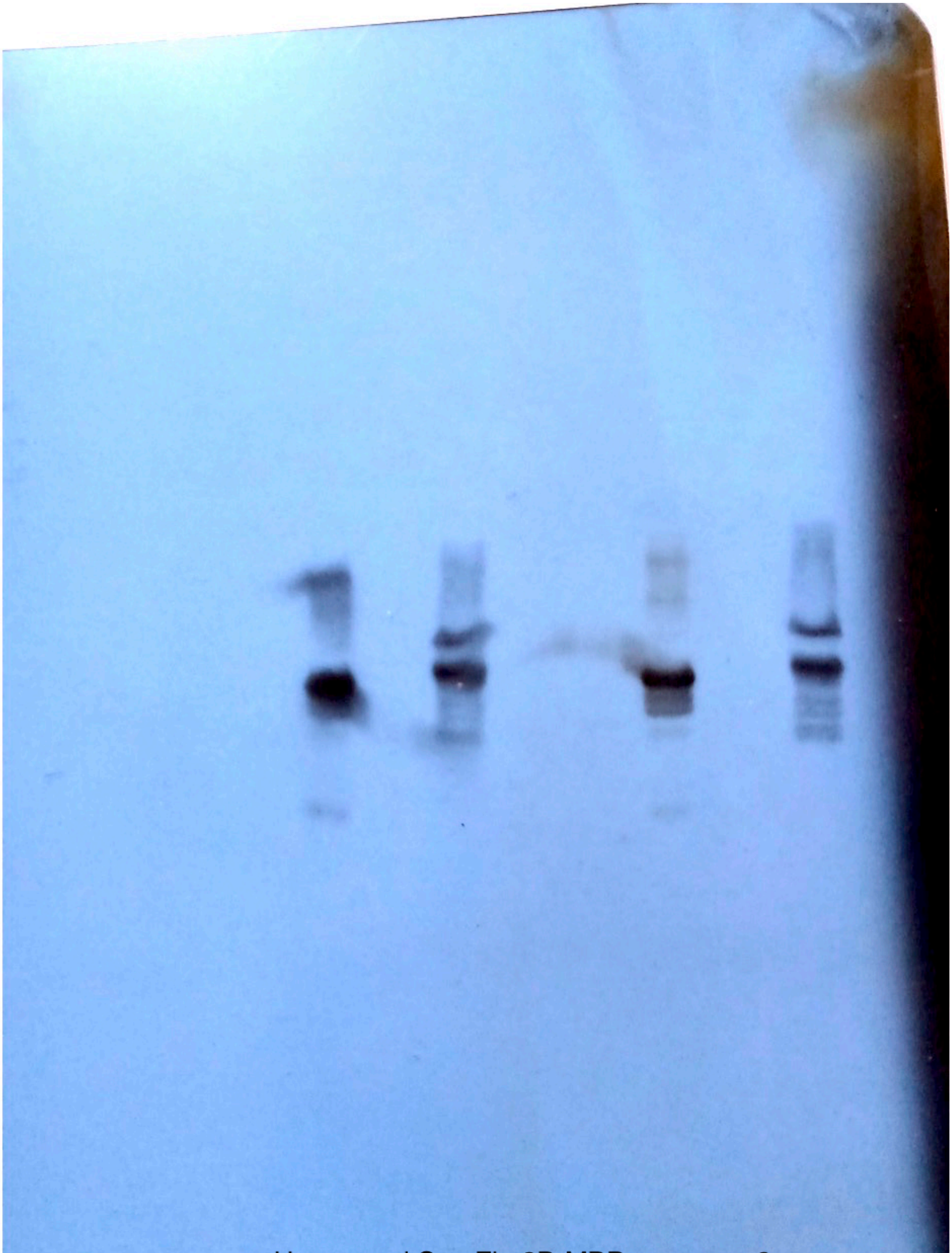

Uncropped Sup Fig 2B-MBP exposure-3
